# Molecular Mechanisms and Probe‐Dependent Effects of Clinically Relevant GABA_A_ Receptor Modulators

**DOI:** 10.1002/ardp.70277

**Published:** 2026-07-03

**Authors:** Marvin Taterra, Yuan Chang‐Halabi, Marcel Bermúdez

**Affiliations:** ^1^ Institute of Pharmaceutical and Medicinal Chemistry Universität Münster Münster Germany; ^2^ GRK 2515, Chemical Biology of Ion Channels (Chembion) Universität Münster Münster Germany

**Keywords:** allostery, ion channel, MD simulations, MDPath, probe dependency

## Abstract

Modulators of γ‐aminobutyric acid type A (GABA_A_) receptor, including benzodiazepines, anaesthetics, and Z‐drugs, act through distinct allosteric mechanisms that remain incompletely understood at the molecular level. Here, we investigate probe‐dependent pharmacology in GABA_A_ receptor modulation by integrating molecular dynamics simulations, deep learning‐based enhanced sampling, dynamic pharmacophores, and our recently developed MDPath approach for mapping allosteric communication networks. We demonstrate that benzodiazepines and Z‐drugs leverage physiological GABA for activity through long‐range allosteric coupling. These modulators bind in the transmembrane domain (TMD), establishing allosteric commmunication to the extracellular domain (ECD) GABA binding sites, stabilizing GABA receptor interactions and explaining their probe‐dependent positive allosteric modulation. In contrast, anaesthetics forms additional interactions that enable direct binding‐driven channel activation independent of GABA, distinguishing ago‐PAMs from PAMs despite binding at the same sites. Remarkably, the flumazenil antidote acts by globally disrupting interdomain allosteric communication, selectively preventing the action of ECD‐dependent modulators (benzodiazepines and Z‐drugs) while sparing anaesthetics' direct effects. Our integrated computational approach reveals the structural determinants of receptor state transitions, providing a framework for understanding probe‐dependent pharmacology in ligand‐gated ion channels with implications for rational drug design.

## Graphical abstract

Combining molecular dynamics, dynamic pharmacophores, and allosteric network analysis, we reveal that benzodiazepines and Z‐drugs stabilize GABA binding through long‐range TMD–ECD allosteric coupling, while anesthetics directly activate the channel via unique L232/I264 contacts, bypassing GABA. Flumazenil selectively reverses GABA‐dependent modulators by globally disrupting interdomain communication, leaving anesthetic‐mediated gating unaffected.

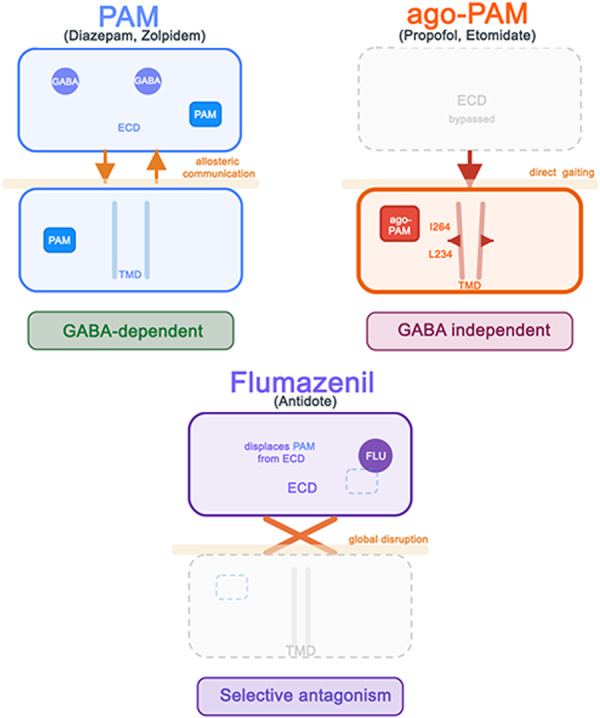

## 1 Introduction

**Figure 1:**
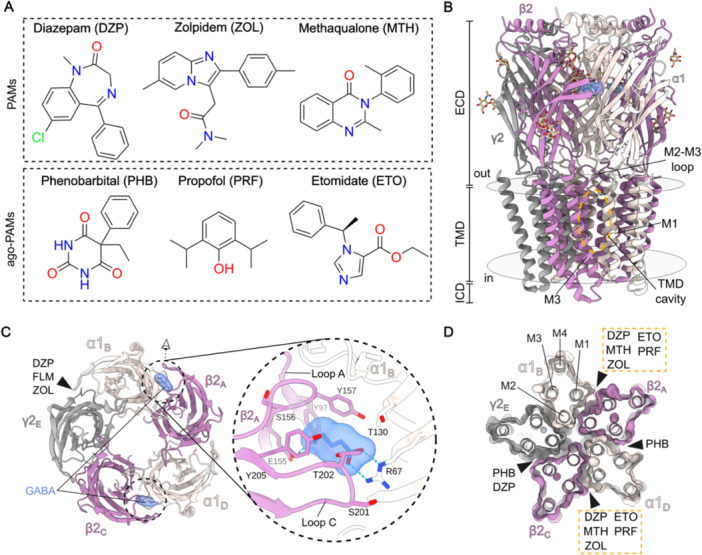
Overview on the GABA_A_ receptor structure and allosteric modulators discussed in this work. (A) Chemical structures for PAMs and agoPAMs discussed in this work. (B) Side view of the heteropentameric GABA_A_ receptor complex oriented in the membrane. Subunits are colored according to identity: α1 subunits in white, β2 subunits in purple, and the γ2 subunit in gray. *N*‐linked glycans are shown in yellow, and bound GABA molecules are shown in light blue. The transmembrane cavity pocket is highlighted by the orange dashed oval. (C) Extracellular view of the principal ligand‐binding sites within the ECD. The locations of the benzodiazepine‐binding site, occupied by diazepam (DZP), flumazenil (FLM), and zolpidem (ZOL), and the orthosteric GABA‐binding sites are indicated. The inset provides a detailed view of the GABA‐binding pocket, highlighting key receptor residues involved in ligand recognition. (D) Extracellular view of the TMD shown in both cartoon and surface representations. The four TMD helices (M1–M4) of an α1 subunit are labeled as an example. The distinct binding regions of the discussed modulators within the TMD are indicated.

The GABA_A_ receptor is one of the principal inhibitory neurotransmitter receptors in the mammalian central nervous system, mediating fast synaptic inhibition through chloride ion conductance [1]. Through this role in controlling neuronal excitability, it represents a critical target for a wide range of clinically relevant drugs (Figure 1A) for different neurological disorders. For example, benzodiazepines, such as diazepam, are widely prescribed for anxiety disorders and epilepsy among other indications [2, 3], while the so‐called Z‐drugs, such as zolpidem, represent an effective treatment for insomnia [4]. General anesthetics, including propofol and etomidate, enable surgical procedures [5, 6], while barbiturates remain valuable in anesthesia and neurocritical care [7, 8], and neurosteroid‐based therapies, such as allopregnanolone, offer new treatment options for postpartum depression [9].

GABA_A_ receptors are pentameric ligand‐gated ion channels assembled from a set of 19 distinct subunits in homo‐ and hetero‐pentameric complexes that are arranged around a central ion‐conducting pore. The β2–α1–β2–α1–γ2 configuration is the predominant form in the brain [10, 11]. (Figure 1B). The receptor architecture reflects a highly allosteric organization: the endogenous agonist GABA binds at β2/α1 subunit interfaces within the extracellular domain (ECD) that drives conformational rearrangements that propagate through the receptor and ultimately open the activation gate located in the M2 helices of the transmembrane domain (TMD). Allosteric modulators can occupy multiple distinct binding sites in both the ECD and in TMD cavities [12, 13, 14]. The latter one is located beneath the M2–M3 loop by the M1 and M2 helices of one subunit and the M3 and M4 helices of the neighbouring subunit (Figure 1B–D). For example, diazepam has a high‐affinity binding site at the α1/γ2 interface in the ECD and has also been suggested to interact with additional, lower affinity sites at inter‐subunit TMD cavities, including β2/α1 and γ2/β2 interface [14]. Because the GABA binding pockets and allosteric binding sites are spatially distinct, effective modulation relies on long‐range allosteric communication, but it remains elusive how this communication works on a mechanistic level, and how specific drug classes divergently interfere with those communication routes.

A broad classification of allosteric GABA_A_ modulators is based on their dependence on the endogenous agonist GABA. Classical benzodiazepines and Z‐drugs, which act as positive allosteric modulators (PAMs), exemplify highly probe‐dependent allosteric modulation [14, 15]. While they elevate GABA‐induced currents, they cannot activate the channel in the absence of the physiological agonist [16, 17]. This probe‐dependence provides an inherent safety mechanism that limits overdose potential [18, 19]. Conversely, barbiturates and general anesthetics function as agonistic PAMs (ago‐PAMs), which can directly activate the receptor without relying on another agonist, while also enhancing GABA responses [18, 20]. Despite decades of clinical use and their profound impact on patient care, the molecular mechanisms underlying allosteric regulation and probe‐dependent pharmacology of GABA_A_ receptor modulators remain partially understood. In particular, the structural and dynamic basis explaining why PAMs strictly require GABA for efficacy, even when some PAMs and ago‐PAMs share overlapping binding pockets, remains unresolved.

Besides modulators that enhance channel‐opening, flumazenil represents a particularly intriguing case of probe‐dependent allosteric antagonism with clinical significance as the antidote for benzodiazepine overdose. It was long believed to act through competitive inhibition at the benzodiazepine binding pocket [21]. However, recent cryo‐electron microscopy (cryo‐EM) structures suggest that flumazenil instead binds in the ECD α1/γ2 interface and exerts effects on the spatially distant TMD benzodiazepine pocket through long‐range allosteric coupling [14]. The differential reversibility of benzodiazepine effects versus propofol at GABA_A_ receptors has direct clinical implications. Recent anesthetic protocols transitioning to benzodiazepines before completion of surgical procedures to leverage flumazenil‐mediated reversal [22] underscore the importance of probe‐dependent pharmacology.

Although recent studies have provided invaluable snapshots of the structure of GABA_A_ receptor (in particular the α1β2γ2 subtype) bound to allosteric modulators [2, 14, 15, 23], the dynamic mechanisms underlying probe‐dependent allosteric communication and the molecular determinants distinguishing PAMs and ago‐PAMs still remain elusive. To address these questions, we combine unbiased molecular dynamics (MD) simulations, deep learning‐based enhanced sampling [24], dynamic pharmacophore (dynophore) analysis [25, 26], and our recent MDPath approach [27] to investigate probe‐dependent effects in GABA_A_ receptor modulation. MDPath analyzes allosteric communication pathways using MD simulations. This open‐source toolkit calculates normalized mutual information on backbone dihedral angles and maps correlated movements to a residue graph for path identification.

We elucidate the mechanisms underlying the allosteric cooperativity between GABA and PAMs, and the molecular basis distinguishing PAMs from ago‐PAMs. We also give new insights into flumazenil's probe‐dependent allosteric antagonism. Our findings provide a mechanistic framework for understanding probe‐dependent pharmacology in this therapeutically important receptor family and offer insights for rational drug design targeting ligand‐gated ion channels. Moreover, we demonstrate the applicability of MDPath in combination with dynophores as a framework for studying probe‐dependent effects.

## 2 Results and Discussion

### 2.1 The Mechanism of GABA Probe Dependence

**Figure 2:**
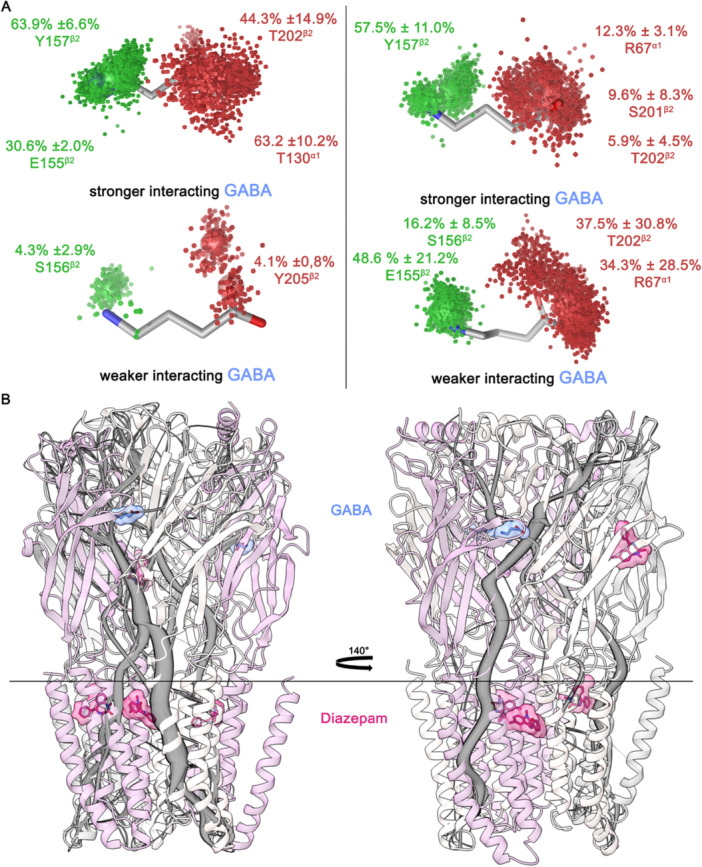
(A) Comparison of representative GABA dynophores computed for a system with only GABA bound to the open state (left) and the same system with diazepam additionally bound (right). Red spheres indicate hydrogen bond acceptor (HBA) interactions, and green spheres indicate hydrogen bond donor (HBD) interactions. The mean interaction frequency and its standard error, derived from triplicate simulations, are shown in the corresponding interaction color. Interacting residues are labeled using the one‐letter amino acid code followed by the residue number, with the subunit indicated as a superscript (β2 or α1). Top panels show the stronger‐interacting GABA; bottom panels show the weaker‐interacting GABA. (B) Allosteric communication pathways (grey lines) originating in the transmembrane domain (TMD) near the diazepam binding residues (diazepam shown in pink) and extending toward the GABA binding pocket (GABA shown in blue) in the extracellular domain (ECD), thereby functionally connecting the two spatially distant binding sites. The right view is rotated by 140° relative to the left.

To understand the impact of diazepam on GABA recognition, we performed unbiased MD simulations of the open‐state channel in both GABA‐bound and GABA/diazepam‐bound conditions. Dynophore analysis of the GABA‐bound receptor revealed divergent binding behavior at the two orthosteric pockets, providing insight into site‐dependent modulation. The GABA molecule positioned at the β2–α1 subunit interface (β2 directly adjacent to the γ2 subunit) exhibited substantially less frequent protein–ligand interactions compared with the molecule at the opposite site. This is consistent with electrophysiological studies demonstrating non‐equivalent properties of the two orthosteric sites [28]. At this weaker interacting GABA‐binding site, dynophore analysis identified only sparse hydrogen bond donor (HBD) and hydrogen bond acceptor (HBA) interactions. In contrast, the GABA molecule at the stronger interacting GABA‐binding site displayed prominent HBD interactions alongside robust HBA interactions (Figure 2A). This divergence in binding stability was further corroborated by spontaneous unbinding of the weaker interacting GABA‐binding site GABA molecule within the final 10 ns of one replica (Supporting Information S1: Section S1 and Figure 2A). Analysis of allosteric communication networks using MDPath revealed that both GABA binding sites serve as regions where allosteric communication paths converge, with paths originating from the ECD and extending deep into the TMD bundle. This suggests that both orthosteric pockets contribute to GABA‐induced channel activation.

Building on this baseline asymmetry of interactions in the different binding pockets, we next examined how diazepam modulates GABA binding. Upon diazepam binding, we observed enhanced GABA interactions at both orthosteric sites. The GABA molecule that initially showed weaker interactions gained substantial HBD and HBA interactions. In one trajectory, a metastable negative ionizable interaction with T202^β2^ (25.6%) was also observed. Rather than arising from a single dominant dynamic change, this enhancement reflected conformational stabilization of the binding pocket (Supporting Information S1: Section S2), rendering it more favorable for protein–ligand interactions (Figure 2A). At the initially strong‐binding site, diazepam induced a redistribution rather than amplification of interactions. Additional metastable negative ionizable interactions with S201^β2^ (29.0%) and T202^β2^ (24.7%) were observed in one trajectory (Supporting Information S1: Section S1.0 and Figure 2A). As the GABA molecule relies primarily on HBA and HBD interactions, which are generally considered to be among the weaker noncovalent contributions to ligand binding affinity, with individual hydrogen bonds typically contributing only −1.5 to −4.7 kcal/mol to the free energy of binding [29], even modest gains in the number or quality of these interactions can meaningfully enhance overall binding strength. Taken together, these results indicate that diazepam strengthens GABA binding at the previously weaker interacting GABA‐binding site while reshaping the interaction landscape at the stronger site.

To understand the structural basis for this allosteric enhancement, we mapped allosteric communication pathways within the receptor. Across all replicas, we identified direct allosteric paths connecting the TMD diazepam binding sites, located below the GABA molecules in the TMD, to the Loop A of the ECD GABA binding pockets (Figure 2B). This long‐range allosteric coupling could explain both the enhanced GABA binding affinity in the presence of diazepam and the reciprocal stabilization of diazepam by GABA, rationalizing the experimentally observed slower dissociation constants for GABA when the receptor is co‐occupied by diazepam [30, 31, 32]. Additional allosteric pathways were identified near the third TMD diazepam binding site. The ECD‐bound diazepam strongly stabilizes the γ2 and adjacent subunits [14], utilizing the Loop A, while the third TMD‐bound diazepam molecule contributes to GABA‐ECD stabilization from within the TMD. Analysis of protein–diazepam interactions revealed that diazepam forms extensive hydrophobic contacts across the different binding pockets, with a notably high frequency of interactions involving methionine side chains. Occasional aromatic and HBA interactions were also detected. Interaction patterns were subunit‐dependent (Supporting Information S1: Section S1.1).

Further investigation into the mechanism of GABA‐dependent PAMs revealed that this allosteric communication and domain stabilization is shared with zolpidem (Supporting Information S1: Section S1.3 and S3) and likely with methaqualone, the infamous predecessor of the benzodiazepines, which binds at the same site and is also strongly GABA‐dependent [23, 33]. Together, these findings point to a shared allosteric architecture that unifies the action of probe‐dependent PAMs through stabilization of the GABA‐bound conformation. Nonetheless, subtle differences in potency between Z‐drugs and benzodiazepines may exist, potentially arising from the third TMD binding site occupied by diazepam but not by zolpidem.

### 2.2 Molecular Determinants Distinguishing PAMs From Ago‐PAMs

The strict GABA dependence of benzodiazepines and Z‐drugs described above raises a fundamental question: why do some modulators require GABA for efficacy, whereas others can directly open the channel? General anesthetics such as propofol and etomidate function as ago‐PAMs, capable of directly activating the receptor in the absence of GABA despite binding to overlapping TMD sites. To understand this functional distinction at the molecular level, we compared the binding modes and dynophores of diazepam, zolpidem, propofol, and etomidate within their shared TMD binding pockets utilizing unbiased MD simulations.

**Figure 3:**
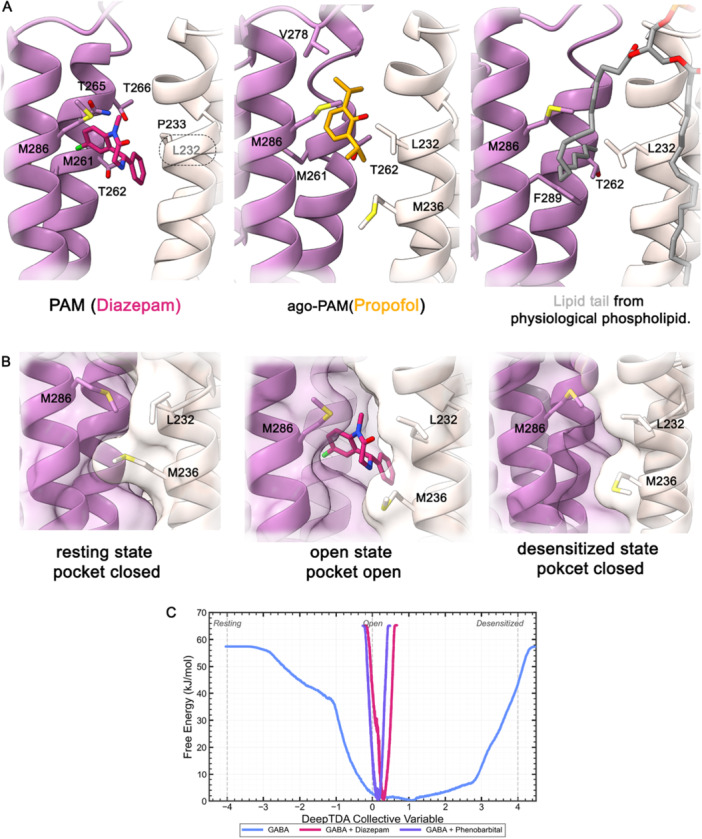
(A) A comparison of the binding of diazepam and propofol to the α1‐β2 pocket. Stable and consistent interactions throughout all replicas of the dynophore analysis are displayed as sticks and labeled accordingly. The ligand, (2S)‐3‐(hexadecanoyloxy)‐2‐[(9Z)‐octadec‐9‐enoyloxy]propyl 2‐(trimethylammonio)ethyl phosphate (grey), shares hydrophobic interaction features with the agoPAMs propofol and etomidate (PDB: 9CRS) [2]. Shown are the lipid's interactions, derived from a pharmacophore generated in LigandScout. Both agoPAMs and the lipid ligand share the interaction with L232, which is not formed by any of the PAMs investigated in this study. (B) The TMD pockets are tightly closed in the resting state and largely closed in the desensitized state (intermediate to resting). In the open state, the pockets are accessible for drug binding, as exemplified by the binding of the diazepam molecule into the open pocket. Prominent large hydrophobic side chains closing the pocket are displayed as sticks and labeled with the corresponding residue numbers. (C) The free‐energy landscape of GABA_A_ alongside the deep‐learned CV for activation/inactivation. Systems in which only GABA is bound are able to transition between the resting, open, and desensitized states. However, those with diazepam or phenobarbital bound to the TMD pockets remain stuck in the open state because state transitions are prevented.

Dynophore analysis revealed that, although propofol and etomidate occupy the same binding pocket and share many interactions with diazepam, they form a unique stabilizing interaction with L232, a feature not observed among the PAMs characterized in this study (Figure 3A). Both anesthetics also form more frequent interactions with pocket residues (Supporting Information S1: Section S1.2 and S1.4). Previous structure–activity relationship (SAR) studies support this finding: metomidate, an etomidate analogue with a methoxy rather than an ethyl ester group, shows markedly reduced potency [34]. Structurally, this change reflects a less favorable interaction with L232 in the etomidate binding pocket. Additionally, an extensive SAR study of etomidate showed that increasing chain bulk led to proportional increases in both GABA‐dependent and non‐GABA‐dependent binding, which scaled linearly [35]. Based on this and our data, the alkyl chain size modulates the ability to interact with L232. Recently, multiple structures have resolved a phospholipid at the α–β and α–γ interfaces that occupies the propofol/etomidate‐equivalent position [2]. Lipids binding to this domain are known to act as PAMs, as they constrict the movement of the TM4 domain and stabilize the open conformation [36]. The endogenous phospholipid resolved with the GABA_A_ receptor from human brain tissue exhibits the same interaction with L232 and shares the core binding interactions that characterize ago‐PAMs (Figure 3A). Thus, ago‐PAMs may mimic the native protein–lipid interactions that are observed in open GABA_A_ receptors. Furthermore, the effects of increasing etomidate's side‐chain length are not necessarily attributable solely to enhanced engagement with residue L232. The concomitant increase in overall hydrophobicity could also enhance membrane partitioning, influence the drug's position within the bilayer, or alter local lipid dynamics [37, 38], thereby shifting the binding equilibrium to favor drug occupancy over endogenous phospholipids that compete for the same pocket. Consistent with the critical role of L232 in ago‐PAM activity, mutagenesis studies have demonstrated that introduction of an L232W substitution abolishes the activity of inhalational anesthetics such as isoflurane and halothane, further underscoring the necessity of this residue for ago‐PAM function [38]. It should be noted, however, that additional mechanisms conferring ago‐PAM activity exist beyond the scope of the canonical PAM binding pocket and, by extension, L232 engagement. Neurosteroids and their derivatives are known to elicit direct receptor activation through distinct binding sites and interaction networks independent of this residue [39].

Beyond L232, both ago‐PAMs interact with I264, a capability lacking in GABA‐dependent PAMs. This interaction causes an inward tilt of the M2 helix, which directly promotes channel opening [14]. Since the M2 helix forms the pore‐lining region, even subtle shifts in its position can greatly affect ion permeation. Consistent with this, previous work showed that etomidate (8.4 Å) and propofol (10.5 Å) produce a wider intrapore diameter at the position opposite the I264‐interacting residue compared with diazepam (5.3 Å), thereby lowering the energy barrier for chloride ion transport through the pore [14]. Direct activation by propofol alone is reportedly up to 20 times greater than co‐activation with GABA, underscoring the potency of this mechanism. As with GABA binding and the classical PAMs, an asymmetrical interaction pattern in the different binding sites is observed in the TMD, where one ago‐PAM molecule binds more favorably than the other. Additionally, earlier studies mutated one of the shared interaction sites for PAMs and ago‐PAMs we identified. Upon introduction of the M286W mutation, propofol's GABA‐dependent activation was eliminated, yet its ability to directly activate the channel was preserved [40, 41]. Together, these ago‐PAM‐specific interactions with L232, I264, and the resulting M2 helix tilt, provide a structural basis for GABA‐independent channel activation.

Extending this analysis to phenobarbital, which binds at the α1_B_‐β2_C_ and γ2_E_‐β2_A_ interface, reveals a similar pattern of asymmetric dynophores in the different binding sites (Supporting Information S1: Section S1.5). Like the other ago‐PAMs, phenobarbital exhibits slightly different binding modes between subunits. In contrast to the previously discussed ligands and especially diazepam (sharing the binding in the γ2_E_‐β2_A_ pocket), phenobarbital displays more extensive HBA‐ and HBD interactions with the binding pocket, driven by its barbituric acid scaffold (Supporting Information S1: Figure S6). Despite this enhanced polar interaction network, the primary driver for barbiturate activity remains the hydrophobic interactions mediated by the bulky C‐5 substituents. For barbiturates, 5,5‐disubstitution with appropriate lipophilic groups (alkyl, aryl) is essential for CNS activity, with optimal activity requiring 6–10 total carbons at the C‐5 position [42]. This interpretation is strongly supported by recent findings showing that compounds featuring cyclization of the ethyl group retain binding affinity through the specific hydrogen bond network, but lose efficacy due to disrupted hydrophobic interactions, thereby acting as silent competitive antagonists [43]. Also, the P228A^β2A^ mutant, which creates an additional hydrophobic side chain adjacent to the ethyl moiety in the binding pocket, enhances GABA‐dependent activation by allowing for more extensive hydrophobic contacts [44]. Collectively, these findings reinforce a model in which hydrophobic engagement of the ethyl group is the principal driver of phenobarbital‐mediated activation.

We next asked why PAMs strictly require GABA. Examining the binding of PAMs on the full receptor equilibrium reveals that they cannot bind to desensitized and resting states to the same extent as to the open state. Recent cryo‐EM structures of the resting (PDB ID: 6X3S) and desensitized (intermediate to resting) (PDB ID: 6 × 40) states have revealed that the necessary TMD pockets close in the non‐open state [14]. Pocket closure observed in the non‐active states is largely caused by the previously shown ligand‐interacting methionine side chains (Supporting Information S1: Section S1 & Figure 3A). In contrast, the pockets that remain accessible, even in the resting state, simply do not contain methionine residues capable of pocket closure (Figure 3B).

To assess this behavior dynamically, we designed a deep collective variable (deepCV) that samples resting, open, and desensitized states, because the corresponding rare transitions are inaccessible to unbiased MD simulations within feasible computational times. In the case of the receptor bound to GABA, we were able to successfully sample the full energetic landscape within only 23.5 ns and 10 walkers, revealing a large barrier of over 50 kJ/mol for the GABA_A_ receptor bound to two GABA molecules to sample the non‐open states. This is in line with previous work demonstrating an 84,000‐fold higher opening rate when the GABA_A_ receptor binds two GABA molecules compared to the apo channel [45]. This shows that the double GABA‐bound state stabilized by PAMs virtually only exists in the active state, being physiologically limited only by the GABA‐binding properties discussed earlier. The single GABA‐bound state is 120 times less active than the double GABA‐bound state [45]. Attempting to replicate those findings with retrained deepCVs revealed that models with GABA+diazepam and GABA+phenobarbital could not leave the open state, with barriers up to 100 kJ/mol, thereby underscoring that state shifts to the states with closed pockets are simply not possible when PAMs are bound (Supporting Information S1: Section S4‐S5 and Figure 3C).

This mechanism is further supported by the observation that receptor opening enhances the binding of diazepam and zolpidem [46, 47]. Previous work has shown that GABA strongly stabilizes the open state, leading to up to a threefold increase in zolpidem binding, while one of the binding pockets in the ECD remains freely accessible. This highlights the sterically constrained nature of the TMD binding pockets and their dependence on channel conformation. A similar pattern was observed for diazepam: at low nanomolar concentrations, it did not interfere with etomidate activity, whereas at higher concentrations it competitively antagonized it [48]. The same study determined diazepam's binding affinity to the etomidate site at approximately 20 μM (to the etomidate‐bound receptor) in the absence of GABA. This is comparable to etomidate's affinity for the inactive receptor (14 μM), but substantially lower than etomidate's affinity for the GABA‐activated state (1.1–2.2 μM) [49]. These findings are further corroborated by the α1 L263S gain‐of‐function mutation, which promotes spontaneous channel opening and demonstrates that diazepam can act as a partial activator, independent of the trigger for activation [50]. Together, these results indicate that a component of PAM activation is directly binding‐driven to the TMD pockets, but its efficacy depends on the receptor's ability to adopt an open state. These findings demonstrate that PAMs depend on GABA‐mediated channel opening to access and stabilize the TMD pockets. In contrast, ago‐PAMs utilize distinct protein–ligand interactions that confer stronger TMD pocket binding, especially via hydrophobic contacts at the cost of diminished ECD interactions, enabling direct channel activation independent of GABA occupancy. Importantly, while GABA‐dependence distinguishes pocket accessibility between these modulator classes, probe‐dependent differences in allosteric communication paths further contribute to their distinct pharmacological profiles.

### 2.3 Selectivity and Mechanism of Flumazenil as an Antidote

The distinction between PAMs and ago‐PAMs has important clinical implications, particularly for the pharmacological reversal of sedation. Flumazenil selectively reverses benzodiazepine effects while sparing anesthetic action, representing another layer of probe‐dependent pharmacology at the GABA_A_ receptor [22]. Contrary to the long‐held belief that flumazenil acts as a competitive antagonist at the benzodiazepine binding site, recent structural studies revealed that flumazenil binds to a distinct allosteric site in the ECD and induces selective closure of the spatially distant β2_C_‐α1_D_ TMD pocket [14]. However, the mechanism underlying this long‐range allosteric coupling and its selectivity for GABA‐dependent modulators has remained unclear. To gain insight into this process, we additionally simulated the GABA_A_ receptor in complex with GABA and flumazenil.

**Figure 4:**
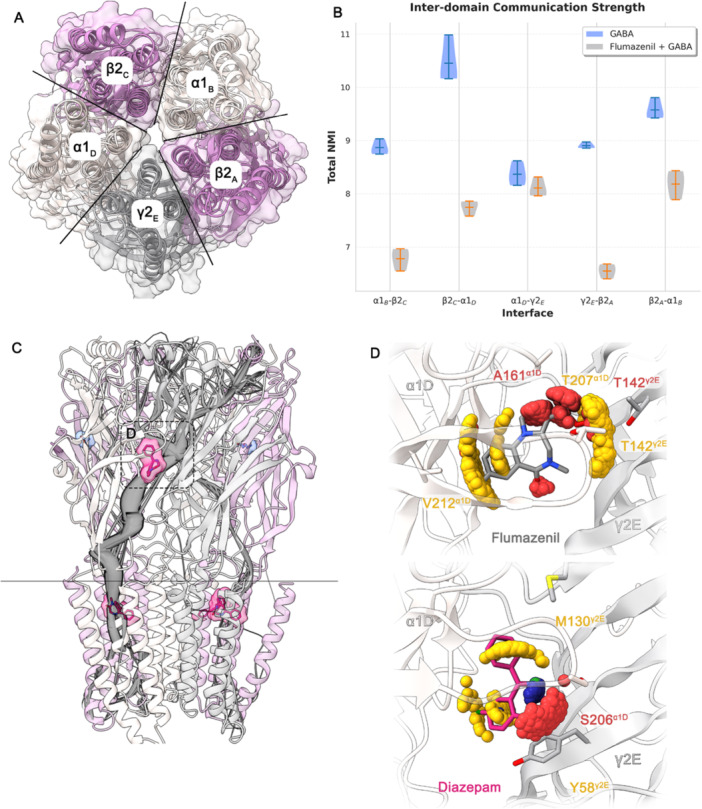
(A) Overview of the subunits and their labeling. (B) Inter‐domain communication strength comparing GABA with GABA and flumazenil. NMI information is pooled from all edges crossing the subunit borders. (C) ECD‐binding diazepam allosteric communication path (gray) reaching through the TMD‐binding diazepam binding pockets. (D) Comparison of the diazepam and flumazenil dynophores both binding in the ECD. The yellow spheres indicate hydrophobic contacts, the blue spheres indicate HBD interactions, and the red spheres indicate HBA interactions. This representation is derived from a ligand‐centered trajectory and aligned onto the first frame. The amino acids forming stable protein–ligand interactions are displayed as sticks and labeled according to the formed interactions following the dynophore coloring scheme.

Analysis of the dynamic protein–ligand interactions revealed that flumazenil forms sparse direct contacts within its ECD binding pocket. Dynophore analysis identified predominantly hydrophobic contacts with Y58^γ2E^ and T142^γ2E^, with one replica showing additional contacts to V203 ^α1D^, T207^α1D^, and HBA interactions with A161 ^α 2A^ (Figure 4D). Although visual inspection suggested potential hydrogen bonding with the nearby histidine H102^α2A^, these interactions did not meet the geometric criteria defined in LigandScout. The interaction with A161^α1D^ and V203 ^α1D^ distinguishes flumazenil from zolpidem and diazepam, which interact in the same ECD pocket. Previous mutational analysis has shown the need for an alanine or another H‐bond donor such as arginine at this position. A bulkier substituent would interfere with the ethyl ester group, which distinguishes flumazenil from the related benzodiazepine scaffold (Figure 4D) [51]. Another striking difference between flumazenil and the PAMs binding in the ECD is that flumazenil is incapable of binding to the TMD pockets. This is because its larger molecular structure, compared with that of the initial benzodiazepine, prevents it from binding to the sterically constrained TMD pockets.

These distinct interaction requirements are further supported by replicating the findings of Dunn et al. [52] using docking. Mutations at H102 (A‐loop) that promote stronger interactions with the α1‐subunit, such as H102Y, H102K, and H102Q, convert flumazenil from an antagonist into a partial agonist. These substitutions reduce the likelihood of interactions within the γ2‐subunit mediated by the imidazole ring and ethyl ester moiety, thereby shifting flumazenil toward a binding mode more similar to classical benzodiazepines. A similar effect is observed with the fluoro‐to‐azide‐substituted analog Ro15‐4513 [52]. The H102E mutation confers partial agonism to this derivative and simultaneously abolishes benzodiazepine binding to the canonical scaffold [52]. This contrast underscores the distinct interaction requirements of flumazenil and its derivatives compared with classical benzodiazepines. Methaqualone, previously shown to act as a silent allosteric modulator at this particular binding pocket, adopts a pose intermediate between the flumazenil and benzodiazepine binding poses [23]. In this position, it is unable to engage the γ2‐subunit interactions characteristic of flumazenil, yet it also fails to establish strong contacts with the A Loop in the α1‐subunit as observed for classical benzodiazepines (Supporting Information S1: Section S6).

While competitive binding at the ECD orthosteric site represents one facet of flumazenil's mechanism, the compound also stabilizes the open state of the receptor, indicating that its pharmacological profile extends beyond simple competitive antagonism. Understanding the allosteric mechanism by which flumazenil binding at the ECD prevents modulation of the TMD pockets is therefore essential for a complete mechanistic picture. Despite these limited direct interactions, flumazenil binding produced pronounced long‐range effects on protein dynamics. Interdomain allosteric network analysis revealed a uniform reduction in communication strengths across all subunit interfaces, leading to reduced protein stabilization (Figure 4A,B). The β2_C_–α1_D_ interface, the site occupied by benzodiazepines, Z‐drugs, propofol, and etomidate, showed the most substantial loss of coupling. This diminished allosteric coupling aligns with the reduced protein resolution previously reported by Kim and colleagues [14], which likely reflects increased structural flexibility or heterogeneity, indicating a consistent mechanistic interpretation across both experimental and computational observations. Importantly, the magnitude of flumazenil's effect on allosteric network communication correlated almost linearly with the number of inter‐domain contacts between subunit residues. This correlation strongly suggests that flumazenil disrupts allosteric communication by altering inter‐domain packing globally, rather than through a single long‐range allosteric path (Figure 4A,B, and Supporting Information S1: Section S7). Overall, this global disruption increases conformational entropy, also providing a favorable entropic contribution to flumazenil binding.

MDPath analysis provides mechanistic insight into how this global disruption translates into selective PAM reversal. In the diazepam‐GABA system, strong allosteric communication paths connect the ECD to the TMD, particularly involving the γ2 subunit (Figure 4C). This bidirectional communication stabilizes both the ECD and TMD binding sites. By globally disrupting interdomain communication, particularly between the α1 and β2 domains, flumazenil effectively abolishes the allosteric coupling required for benzodiazepine and Z‐drug activity, thereby preventing their PAM effects. MDPath analysis of the flumazenil‐bound state further supports this mechanism, revealing a notable absence of major allosteric pathways originating from the flumazenil binding region (Supporting Information S1: Figure S15). A key distinction from diazepam is the lack of allosteric communication crossing the A‐loop, consistent with flumazenil's lack of interaction with this region. Previous structural work demonstrated that substituting ECD‐bound diazepam with flumazenil causes dissociation of TMD‐bound diazepam at the adjacent β2_C_‐α1_D_ site and subsequent pocket closure [14], a direct consequence of this abolished communication.

Essentially, this mechanism explains the probe‐dependency of flumazenil with regard to the PAM versus ago‐PAM activity. Flumazenil's reversal of benzodiazepine and Z‐drug activity operates through a dual mechanism: direct competition at the shared ECD binding site, displacing PAMs from their pocket, combined with the global disruption of interdomain allosteric communication required for their TMD‐mediated effects. In contrast, ago‐PAMs such as propofol and etomidate are optimized for the TMD pockets through additional stabilizing interactions, particularly with L232 and I264, that enable direct channel activation independent of ECD occupancy and the ECD‐TMD allosteric communication pathways. Because flumazenil neither competes at the TMD binding sites nor can abolish these direct gating interactions, ago‐PAMs maintain their activity through mechanisms that bypass the disrupted allosteric pathways. Thus, while flumazenil abolishes the GABA‐dependent positive modulation mediated by benzodiazepines and Z‐drugs, it cannot reverse the direct gating effects of anesthetics, explaining the clinical observation that flumazenil reverses benzodiazepine sedation but not propofol anesthesia [22].

## 3 Conclusion

In this work, we elucidated the molecular mechanisms underlying probe‐dependent pharmacology at the GABA_A_ receptor. By integrating MDPath allosteric network analysis, dynophore profiling, and deep‐learning–based CV‐enhanced sampling, we elucidated how structurally distinct modulators produce divergent functional outcomes despite sharing overlapping binding sites.

We demonstrated that benzodiazepines and Z‐drugs enhance the stability of receptor‐GABA interactions at the orthosteric binding pockets through long‐range allosteric communication originating from the TMD and propagating to the ECD. This allosteric coupling increases GABA affinity and promotes a strongly stabilized GABA‐bound open state, a finding further validated by enhanced sampling, which revealed barriers of 100 kJ/mol against transitions to non‐open conformations.

In contrast, flumazenil acts as a global allosteric disruptor, weakening inter‐domain communication uniformly across the receptor. The magnitude of its effect scaled linearly with the number of interdomain contacts, indicating broad disruption of inter‐domain packing rather than interruption of a single discrete allosteric pathway. Combined with direct competition at the shared ECD binding site, this mechanism selectively abolishes the activity of GABA‐dependent modulators while sparing direct gating processes.

General anesthetics such as propofol and etomidate evade flumazenil's antagonism through enhanced protein–ligand contacts within the TMD pockets—particularly with L232 and I264, that resemble those formed by endogenous phospholipids resolved in the GABA_A_ receptor isolated from human brain. These interactions enable direct, GABA‐independent channel activation, distinguishing ago‐PAMs from classic PAMs. Crucially, the same TMD‐optimized binding that confers direct gating ability also renders these ligands insensitive to flumazenil's disruption of ECD‐TMD allosteric communication.

These findings carry implications for drug design. Because the ability to bind lipids within pore‐lining TMD domains is conserved across diverse ion channels [53], direct targeting of these pockets may offer greater efficacy than relying solely on allosteric mechanisms mediated through the ECD, as such ligands would not be limited by ECD competition or GABA dependence.

The computational approach presented here proved highly effective in capturing complex allosteric phenomena across multiple receptor states. Notably, the domain‐based analysis newly integrated in this work represents an extension beyond the original MDPath workflow, enabling a more granular dissection of inter‐domain communication pathways. Importantly, the integrated pipeline of MDPath, dynophore analysis, and deep‐learning–based enhanced sampling does not require system‐specific GABA_A_ receptor inputs, suggesting broad transferability to other ligand‐gated ion channels and multi‐subunit receptors.

Several limitations of the present model should nonetheless be noted. Classical MD with the CHARMM36m/CGenFF force fields, together with the accessible sampling timescales, cannot fully reproduce the physiological timescales of GABA_A_ receptor gating and ligand (un)binding, and the DeepTDA collective variable (CV) is by construction anchored on three experimentally resolved endpoints (resting, open, and a desensitized‐like state). Furthermore, the approach requires prior structural knowledge of the specific receptor states under investigation. In addition, the present analysis was based on a single subunit composition from one cryo‐EM ensemble, and alternative stoichiometries or structures resolved under different conditions might each warrant individual investigation. The mechanistic proposals made here, in particular the predicted allosteric contact residues, the probe‐dependent reversal by flumazenil, and the distinction between direct and indirect gating are consistent with the mutational and electrophysiological data available in the literature, but prospective experimental validation of newly predicted residues, for example by site‐directed mutagenesis combined with electrophysiological recordings, remains a natural next step beyond the scope of the present work. Despite these limitations, this framework opens new avenues for rational drug design and for systematically dissecting allosteric mechanisms in complex pharmacological targets.

## 4 Materials and Methods

### 4.1 System Setup

Structures (bicuculline‐bound (PDB: 6X3S) [14], the GABA‐bound (PDB: 6X3Z) [14], the picrotoxin‐bound (PDB: 6×40) [14], flumazenil–GABA co‐bound (PDB: 6X3U) [14], diazepam–GABA co‐bound (PDB: 6X3X) [14], phenobarbital‐GABA co‐bound (PDB:6X3W) [14], propofol‐GABA co‐bound (PDB:6X3T) [14], etomidate‐GABA co‐bound (PDB: 6X3V) [14] and zolpidem‐GABA (PDB: 8DD2) [15]) were retrieved from the PDB (RCSB.org) [54] and prepared using MOE 2024.0601 [52]. Additional antibodies, waters, and ions were removed. Missing atoms were added, and atom clashes along with Ramachandran outliers were minimized using the AMBER14:ETH forcefield in MOE [55]. No Ramachandran outliers remained. All systems were automatically protonated at pH 7.4 before simulation. (For backward compatibility, we retained the numbering scheme used in earlier studies by adopting the original PDB numbering. This ensures direct comparability with previous work. Supporting Information S1: Section S8 shows a direct alignment of the wild‐type human sequence with this numbering scheme.).

### 4.2 Unbiased MD Simulations

MD simulations of all systems were prepared using CHARMM‐GUI [56]. Systems were embedded in a POPC lipid bilayer, and chain endings were capped. All systems were solvated in a cubic TIP3P water box containing 0.15 M NaCl with the dimensions 110 × 110 A with an increased 35 A water padding. This yielded final box dimensions of 110 × 110 × 183 A^3^ for the simulations. Simulations were performed using GROMACS 2024.3 [57] patched with plumed v. 2.9 employing the CHARMM36m forcefield and CGenFF for ligands [58, 59, 60]. Simulations were run on NVIDIA GeForce RTX 4090 GPUs. Before production, the system was minimized using CHARMM‐GUI, and the six‐step minimization and equilibration provided by CHARMM‐GUI [56] was followed.

The system was first minimized using the steepest descent algorithm for 5000 steps, or until the maximum force on any atom dropped below 1000.0 kJ/mol/nm. The neighbor list was updated every 10 steps with a cutoff distance of 1.2 nm for both van der Waals and Coulomb interactions. The Verlet cutoff scheme was used, applying a force‐switch modifier for van der Waals forces between 1.0 and 1.2 nm. Electrostatic interactions were treated using the Particle Mesh Ewald (PME) method [61], with a 1.2 nm real‐space cutoff separating the short‐range (direct‐space) and long‐range (reciprocal‐space) contributions. Positional restraints were applied to the backbone (4000 kJ/mol/nm^2^), side chains (2000 kJ/mol/nm^2^), and lipids (1000 kJ/mol/nm^2^), while dihedral restraints were set at 1000 kJ/mol/rad^2^. Hydrogen bonds were constrained using the LINCS algorithm [62].

After minimization, the system was equilibrated in six steps to gradually release the restraints. The first step used strong positional restraints on the backbone, side chains, and lipids (4000, 2000, and 1000 kJ/mol/nm^2^) and dihedral restraints of 1000 kJ/mol/rad^2^. Restraints were progressively reduced in subsequent steps to 2000, 1000, 500, 200, 50, and finally 0 kJ/mol/nm^2^, depending on the atom type. The first two equilibration runs (125 ps each) used a 0.001 ps timestep without pressure coupling. From the third step onward, semi‐isotropic pressure coupling was applied using a C‐rescale barostat (1.0 bar, 5.0 ps coupling constant, 4.5 × 10^–5^ bar^–1^ compressibility), and the timestep was increased to 0.002 ps for the final three runs of 500 ps each [63]. The temperature was maintained at 303.15 K using a v‐rescale thermostat with a 1.0 ps coupling constant applied to solute, membrane, and solvent groups [64]. In total, the equilibration spanned 1.875 ns, during which all restraints were gradually relaxed until the system reached a stable, equilibrated state.

MD simulations were performed in triplicate for each system, with trajectories of 200 ns recorded per run, corresponding to 8000 frames each, when used for model training and 2000 frames otherwise. The simulated systems included the bicuculline‐bound receptor (PDB: 6X3S), the GABA‐bound receptor (PDB: 6X3Z), the picrotoxin‐bound receptor (PDB: 6×40), flumazenil–GABA co‐bound receptor (PDB: 6X3U) and diazepam–GABA co‐bound receptor (PDB: 6X3X), phenobarbital‐GABA co‐bound receptor (PDB:6X3W), propofol‐GABA co‐bound receptor (PDB:6X3T), etomidate‐GABA co‐bound receptor (PDB: 6X3V), and zolpidem‐GABA (PDB: 8DD2). To allow the reader to assess the quality of the MD simulations used for the downstream analyses, a compilation of dynophore analyses, RMSD time courses for the whole protein, the individual domains discussed in this work (ECD and TMD), as well as for the main ligand and co‐bound GABA (where present), is provided in Supporting Information S1: Section S9.

### 4.3 Deep Learning CV Construction

Structures derived from a consistent experimental framework were used to define the CV states. The resting (closed) conformation was taken from the bicuculline‐bound complex (PDB: 6X3S) and the intermediate, partially desensitized conformation from the picrotoxin‐bound receptor (PDB: 6×40). These structures were then used to define the fixed CV states [14]. The open (active) state was defined flexibly for each model. For the open ensemble, the GABA‐bound receptor (PDB: 6X3Z) was used as a base, alongside the diazepam–GABA co‐bound receptor (PDB: 6X3X), the phenobarbital–GABA co‐bound receptor (PDB: 6X3W).

All Cα‐α cross‐distances (∼1.4 M descriptors) were computed using MDAnalysis (v. 2.9.0) [65, 66], NumPy, and pandas, parallelized via multiprocessing. Feature order was harmonized and stored in.npz format, yielding 24,000 data points with over 1.4 M features. Distance values for specific residue pairs were re‐indexed to a shared canonical ordering (based on alphabetically sorted residue‐label pairs) to ensure a unified format corresponding to the input array representation across all three conformational states; no numerical scaling of the distance values was applied. Calculating the distances in this way reduced the time required for the update step during the MD simulation, enabling more efficient use of the hardware.

To identify the most discriminative structural features, one‐way analysis of variance (ANOVA) was performed on each Cα–Cα distance across the three states. The F‐statistic was computed for each feature, and the top 10,000 features with the highest F‐values were retained, representing distances that showed the greatest variance between conformational states while maintaining within‐state consistency.

A Deep Targeted Discriminant Analysis (DeepTDA) neural network was trained to learn a CV distinguishing the three conformational states [24]. Hyperparameter optimization was performed using Optuna [67] with 25 trials on the full dataset (72,000 frames total: 24,000 per state across triplicates), employing median pruning to terminate unpromising configurations early. The search space included network depth (2–5 layers), layer widths (128–2048 neurons), activation functions (ReLU and ELU), learning rate (10^−5^–10^−2^), and weight decay (10^−6^–10^−3^). Target centers were set at −4.0, 0.0, and 4.0 for the resting, active, and desensitized‐like states, respectively, with a target sigma of 0.5 to allow controlled separation in the learned CV space.

The optimal architecture for each model was used for final model training. The model was trained for a maximum of 500 epochs with early stopping implemented to halt training if the loss did not improve for 50 consecutive epochs. Batch size was dynamically adjusted based on available GPU memory to maximize training efficiency.

All computations were performed on a single NVIDIA GeForce RTX 2070 Super GPU with an overclocked AMD Ryzen 9 9950X3D processor. The final model was implemented using the mlcolvar [68] package with the 10,000 ANOVA‐selected features as input (Supporting Information S1: Section S4).

Gradient‐based attribution methods were applied to identify the most important Cα–Cα distances for state classification. Using a stratified subset of 2000 samples, we computed both standard input gradients and integrated gradients [69] with 30 interpolation steps from a zero baseline. Feature importance scores were calculated as the mean of both methods and used to rank all 10,000 input features. Batch processing (batch sizes: 50 for gradients, 25 for integrated gradients) was employed to manage computational memory. The top 100 features were selected for structural interpretation.

### 4.4 Analysis of Long‐Range Allosteric Effects

All replicas from the Flumazenil‐bound complex, GABA‐bound complex, GABA +zolpidem and GABA + diazepam‐bound complex were analyzed using MDPath. To avoid ambiguity arising from the symmetry of the GABA receptor subunits, all residues were renumbered sequentially starting from 1 across all replicas. The analysis was conducted using the default MDPath parameters with a change in saving the top paths from 500 in the original settings to 2500. Visualization of the resulting allosteric pathways was performed in ChimeraX [70], displaying spline representations overlaid on the topology of the first trajectory frame.

### 4.5 Domain‐Based Allosteric Communication

To quantify the strength of dynamic coupling within and between subunit domains, a systematic normalized mutual information analysis using the pre‐computed graphs was performed. The protein structure was segmented into functional domains according to a predefined configuration file that specified residue ranges for each subunit domain. For each domain, we extracted all constituent residues based on their sequential numbering. Let *D* = {*D*_1_, *D*_2_,…, *D*_n_} be the set of functional domains, where each domain *D*_i_ contains residues:

$$D_{i}=\{r_{i,1},r_{i,2},\ldots ,r_{i,k_{i}}\},$$

where *k_i_* is the number of residues in domain *D_i_*. We then classified all edges in the mutual information network into two categories: intra‐domain edges, connecting residues within the same domain, and inter‐domain edges, connecting residues across different domains. For a mutual information network with edge set E, each edge *e_jk_* connecting residues *r_j_* and r_k_ with weight *w_jk_* (the NMI value) was classified as:

**Intra‐domain edges:**

$$E_{\text{intra}}^{\left(i\right)}=\{e_{\mathrm{jk}}\in E|r_{j}\in D_{i}\wedge r_{k}\in D_{i}\},$$

**Inter‐domain edges:**

$$E_{\text{inter}}^{\left(i,l\right)}=\{e_{\mathrm{jk}}\in E|\left(r_{j}\in D_{i}\wedge r_{k}\in D_{l}\right)\vee \left(r_{j}\in D_{l}\wedge r_{k}\in D_{i}\right),i\neq l\}.$$

For each edge, we extracted the NMI, which quantifies the correlation in conformational dynamics between residue pairs. We calculated both the total NMI (sum of all edge weights) and average NMI (mean edge weight) for each domain individually and for each pair of domains. For individual domains (intra‐domain coupling), the total NMI was calculated as:

$${\text{NMI}}_{\text{total}}^{\left(i\right)}=\sum_{e_{\mathrm{jk}}\in E_{\text{intra}}^{\left(i\right)}}w_{\mathrm{jk}},$$

and the average NMI as:

$${\text{NMI}}_{\text{avg}}^{\left(i\right)}=\frac{1}{\left|E_{\text{intra}}^{\left(i\right)}\right|}\sum_{e_{\mathrm{jk}}\in E_{\text{intra}}^{\left(i\right)}}w_{\mathrm{jk}},$$

where $\left|E_{\text{intra}}^{\left(i\right)}\right|$ is the number of intra‐domain edges in domain D_i_. For domain pairs (inter‐domain coupling), the total NMI was calculated as:

$${\text{NMI}}_{\text{total}}^{\left(i,l\right)}=\sum_{e_{\mathrm{jk}}\in E_{\text{inter}}^{\left(i,l\right)}}w_{\mathrm{jk}},$$

and the average NMI as:

$${\text{NMI}}_{\text{avg}}^{\left(i,l\right)}=\frac{1}{\left|E_{\text{inter}}^{\left(i,l\right)}\right|}\sum_{e_{\mathrm{jk}}\in E_{\text{inter}}^{\left(i,l\right)}}w_{\mathrm{jk}},$$

where $\left|E_{\text{inter}}^{\left(i,l\right)}\right|$ is the number of inter‐domain edges between domains *D_i_* and *D_l_*. The total NMI provides a measure of the overall dynamic coupling strength, accounting for both the number and strength of interactions, while the average NMI reflects the typical interaction strength independent of network density.

### 4.6 Enhanced Sampling Simulations

Enhanced sampling simulations were performed using the trained DeepTDA model as a CV. The model was evaluated and biased every 500 timesteps through the libtorch interface via PLUMED to access the PyTorch model. The CV calculation utilized only the 10,000 ANOVA‐selected features, maintaining the standardized array ordering established during model training. Biasing was applied using the On‐the‐Fly Probability Enhanced Sampling with metadynamics‐like target distribution (OPES) method with a maximum energy barrier set to 60 kJ/mol. The simulations employed a multiple‐walker scheme with 10 walkers, each running for approximately 24 ns. The total simulation time was constrained by the maximum cluster wall time. After thorough analysis of the OPES‐ZED and RCT data, both values were found to plateau within this timeframe, indicating adequate sampling across the deep CV space. The following systems were investigated to characterize conformational behavior along the activation pathway: GABA‐bound (PDB: 6X3Z, 24 ns), diazepam–GABA co‐bound (PDB: 6X3X, 22 ns), phenobarbital–GABA co‐bound (PDB:6X3W, 15 ns).

### 4.7 Reweighting of Biased Trajectories

To obtain unbiased free energy estimates, a reweighting procedure was applied to correct for the bias introduced during the enhanced sampling simulations. This approach follows the method described by Invernizzi et al. [71]. Data were collected from multiple walkers, each producing a COLVAR file containing time‐series data for the reaction coordinate, the bias potential, and other relevant quantities. The reweighting factor for each sampled configuration was computed as:

$$w_{i}=e^{\beta V_{i}},$$

where $\beta ={\left(k_{B}T\right)}^{-1}$ is the inverse thermal energy, and *V_i_* is the bias potential applied at configuration _*i*_. The weights were normalized to construct a proper probability distribution. A kernel density estimation method was then used to compute the weighted probability density function of the reaction coordinate. The corresponding free energy profile was obtained via:

$$F\left(x\right)=-k_{B}T\ln P\left(x\right),$$

where P(x) is the reweighted probability density of the deep CV. To estimate uncertainties, bootstrap resampling was applied to the reweighted dataset. A total of 100 bootstrap replicas were generated, from which the standard error at each value of the reaction coordinate was calculated.

### 4.8 Coding and Writing, Resources

Coding assistance was provided by Claude [Anthropic PBC,San Francisco, CA]. Grammar, spelling, punctuation and tone were edited with ChatGPT [OpenAI, L.L.C., San Francisco, CA], Claude[Anthropic PBC, San Francisco, CA] and DeepL [DeepL SE, Cologne, Germany].

## Data and Software Availability

The MDPath source code is publicly available at https://github.com/wolberlab/mdpath. and can be installed through the Python Package Index (PyPI) and conda including the function for the domain‐based analysis. The code used to train deepCV is available at: https://github.com/MarvinTaterra/Molecular-Mechanisms-and-Probe-Dependent-Effects-of-Clinically-Relevant-GABAA-Receptor-Modulators.git. PDB structures are available from the Protein Data Bank (https://www.rcsb.org) [51] and include: 6X3S [14], 6X3Z [14] 6×40 [14], 6X3V [14], 6X3T [14], 6X3U [14], 6X3W [14], 8DD2 [15], 9CRS [2], 8VQY [23] and 6X3X [14]. The simulation software GROMACS is freely and publicly available (https://github.com/gromacs/gromacs), as is PLUMED, (https://github.com/plumed *)*. MD trajectories generated and analyzed in this manuscript are available from *ScienceDB*: https://doi.org/10.57760/sciencedb.27672 including the plumed specific inputs.

## Conflicts of Interest

The authors declare no conflicts of interest.

## Supporting information

Supporting File

## Data Availability

The data that support the findings of this study are openly available in ScienceDB at https://doi.org/10.57760/sciencedb.27672.
