# Supplementary material for: Molecular Mechanisms and Probe‐Dependent Effects of Clinically Relevant GABAA Receptor Modulators
Source: Arch Pharm (Weinheim). 2026 Jul 3;359(6):e70277. doi: 10.1002/ardp.70277 (PMC13330556; doi:10.1002/ardp.70277)
Supplement: Supplementary file 1 — Supporting File [file ARDP-359-e70277-s001.pdf]

Supplementary Information for "Molecular Mechanisms and Probe-Dependent Effects of Clinically Relevant $GABA_A$ Receptor Modulators"

Marvin Taterra,* Yuan Chang-Halabi,* and Marcel Bermúdez*

Universität Münster, Institute of Pharmaceutical and Medicinal Chemistry, Correnssstr. 48, 48149 Münster, Germany

E-mail: mtaterra@uni-muenster.de; yuan.chang@uni-muenster.de; m.bermudez@uni-muenster.de

Phone: +49 251 83 32272. Fax: +49 251 83 32211

Supplementary Information Contents

S1 Dynophore Analysis **3**

S1.0 GABA 3

S1.1 Diazepam 4

S1.2 Propofol 6

S1.3 Zolpidem 7

S1.4 Etomidate 10

S1.5 Phenobarbital 11

S2 Diazepam GABA probe dependence **13**

S3 Zolpidem GABA probe dependence **14**

S4 Explainable AI of deep-learned $\mathbf{GABA_A}$ State Transition Pathways **15**

S5 DeepTDA Details **18**

Hyperparameter Optimization 18

S6 H101 mutations **19**

S7 Flumazenil allosteric communication **21**

S8 Numbering and alignment **23**

S9 Validation and Convergence of MD Simulations **24**

List of Supplementary Information Figures **33**

List of Supplementary Information Tables **38**

References **38**

S1 Dynophore Analysis

S1.0 GABA

Summary of receptor–ligand interactions identified using *Dynophores*.[1–6] For each binding pocket, key residues, interaction types, and interaction frequencies are listed, comparing the GABA-bound receptor with the same system having additional diazepam bound.

**Binding Site**	**Residue**	**Type**	**GABA**	**GABA + Diazepam**
Weak	S156$^{\beta2}$	HBD	10.1, 1.9, 1.0	17.6, 30.3, 0.8
	Y205$^{\beta2}$	HBA	5.8, 3.5, 3.1	—
	E155$^{\beta2}$	HBD	—	75.9, 62.2, 7.7
	T202$^{\beta2}$	HBA	—	15.0, 0.4, 97.2
	R67$^{\alpha1}$	HBA	—	12.0, 90.8, 0
	T202$^{\beta2}$	NI	—	25.6
Strong	Y157$^{\beta2}$	HBD	67.4, 73.1, 51.1	78.7, 52.3, 41.5
	E155$^{\beta2}$	HBD	29.7, 34.4, 27.8	—
	T130$^{\alpha1}$	HBA	80.2, 45.0, 64.5	—
	T202$^{\beta2}$	HBA	27.9, 74.0, 31.1	2.6, 0.4, 14.6
	R67$^{\alpha1}$	HBA	—	13.5, 6.5, 16.9
	S201$^{\beta2}$	HBA	—	1.9, 0.8, 26.0
	S201$^{\beta2}$	NI	—	29.0
	T202$^{\beta2}$	NI	—	24.7

S1.1 Diazepam

Table S1: Summary of receptor-ligand interactions identified using *Dynophores*.[1–6] Key binding site residues and interaction types are listed for each binding pocket.

**Pocket**	**$GABA_A$ Residues**	**Interaction Types**
ECD	$Y58^{\gamma 2E}$, $M130^{\gamma 2E}$	Hydrophobic
	$Y58^{\gamma 2E}$	Aromatic
	$S206^{\alpha 1D}$	H-bond acceptors
β2A-α1B	$T262^{\alpha 1B}$, $M261^{\alpha 1B}$, $T266^{\alpha 1B}$ T265β1A, $M286^{\alpha 1B}$	Hydrophobic
	$P233^{\beta 1A}$	H-bond acceptors
	$P233^{\beta 1A}$	Aromatic
γ2E-β2A	$V300^{\gamma 2E}$, $M296^{\gamma 2E}$, $V293^{\gamma 2E}$ $M227^{\beta 2A}$	Hydrophobic
	$T277^{\gamma 2E}$	H-bond acceptors
	$Q224^{\beta 2A}$	Aromatic
α1D-β2C	$M286^{\beta 2C}$, $T265^{\alpha 1D}$, $M236^{\alpha 1D}$, $M261^{\beta 2C}$	Hydrophobic

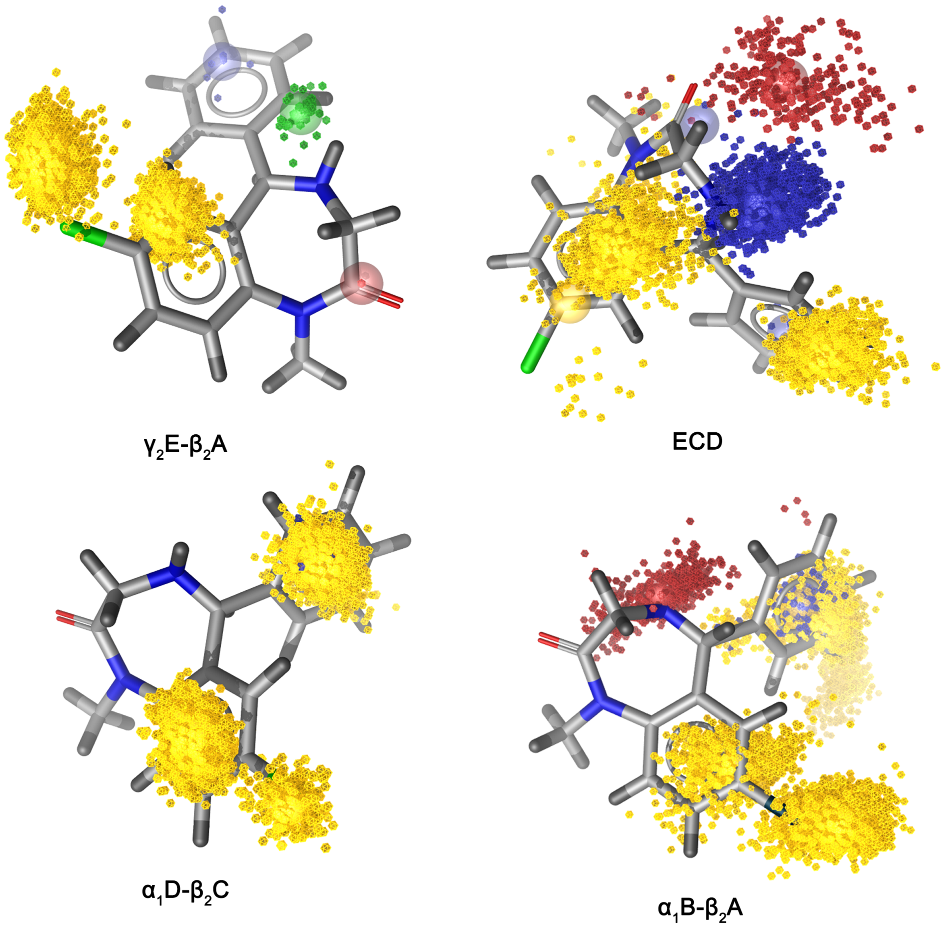

Figure S1: Point cloud representation of the diazepam dynophore. We can see concise point clouds proving the stability of the ligand during the MD simulation. Each point within a cloud represents an interaction in one frame, with blue indicating positive ionizable interaction, red hydrogen bond acceptors and yellow hydrophobic contacts.

S1.2 Propofol

Table S2: Summary of receptor-ligand interactions identified using *Dynophores*.[1–6] Key binding site residues and interaction types are listed for each binding pocket.

**Pocket**	**$GABA_A$ Residues**	**Interaction Types**
β2A-α1B	$M236^{\alpha 1B}$, $L232^{\alpha 1B}$, $M286^{\alpha 1B}$ $T262^{\alpha 1B}$, $V278^{\beta 2A}$, $M261^{\alpha 1B}$	Hydrophobic
α1D-β2C	$I264^{\beta 2C}$	Hydrophobic
	$N265^{\beta 2C}$	HBD

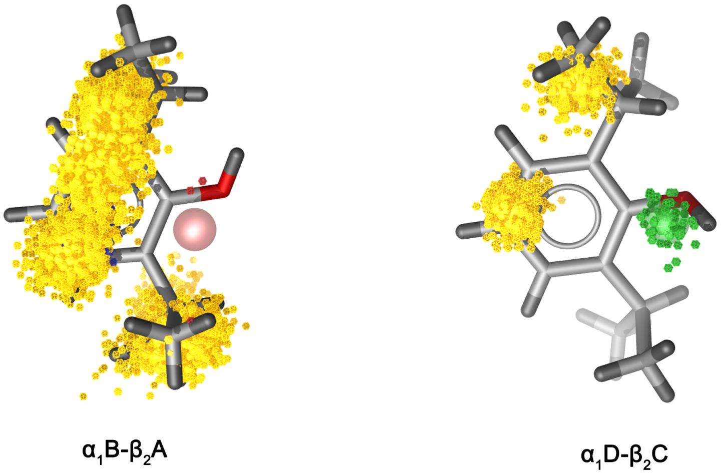

Figure S2: Point cloud representation of the propofol dynophore. We can see concise point clouds proving the stability of the ligand during the MD simulation. Each point within a cloud represents an interaction in one frame, with green hydrogen bond donors, red hydrogen bond acceptors and yellow hydrophobic contacts.

S1.3 Zolpidem

Table S3: Summary of receptor-ligand interactions identified using *Dynophores*.[1–6] Key binding site residues and interaction types are listed for each binding pocket.

Pocket	$GABA_A$ Residues	Interaction Types
ECD	$Y58^{\gamma 2E}$, T142, $M130^{\gamma 2E}$	Hydrophobic
	$S206^{\beta 2A}$, $T207^{\alpha 1D}$	HBA
	$S206^{\alpha 1D}$	Aromatic
α1D-β2C	$M236^{\alpha 1D}$, $T265^{\beta 2C}$ $M286^{\alpha 1D}$	Hydrophobic
	$V290^{\beta 2C}$	HBA
α1B-β2A	$M286^{\alpha 1B}$, $T262^{\alpha 1B}$, $M236^{\alpha 1B}$	Hydrophobic
	$P233^{\beta 1A}$	Aromatic

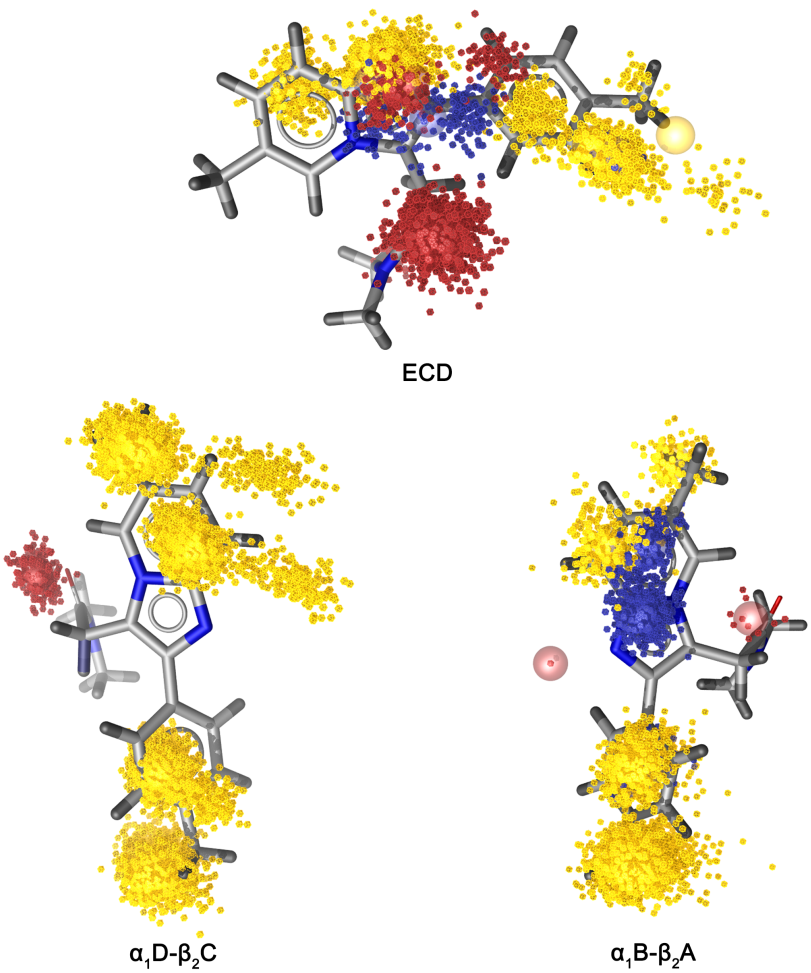

Figure S3: Point cloud representation of the zolpidem dynophore. We can see concise point clouds proving the stability of the ligand during the MD simulation. Each point within a cloud represents an interaction in one frame, with blue indicating positive ionizable interaction, red hydrogen bond acceptors and yellow hydrophobic contacts.

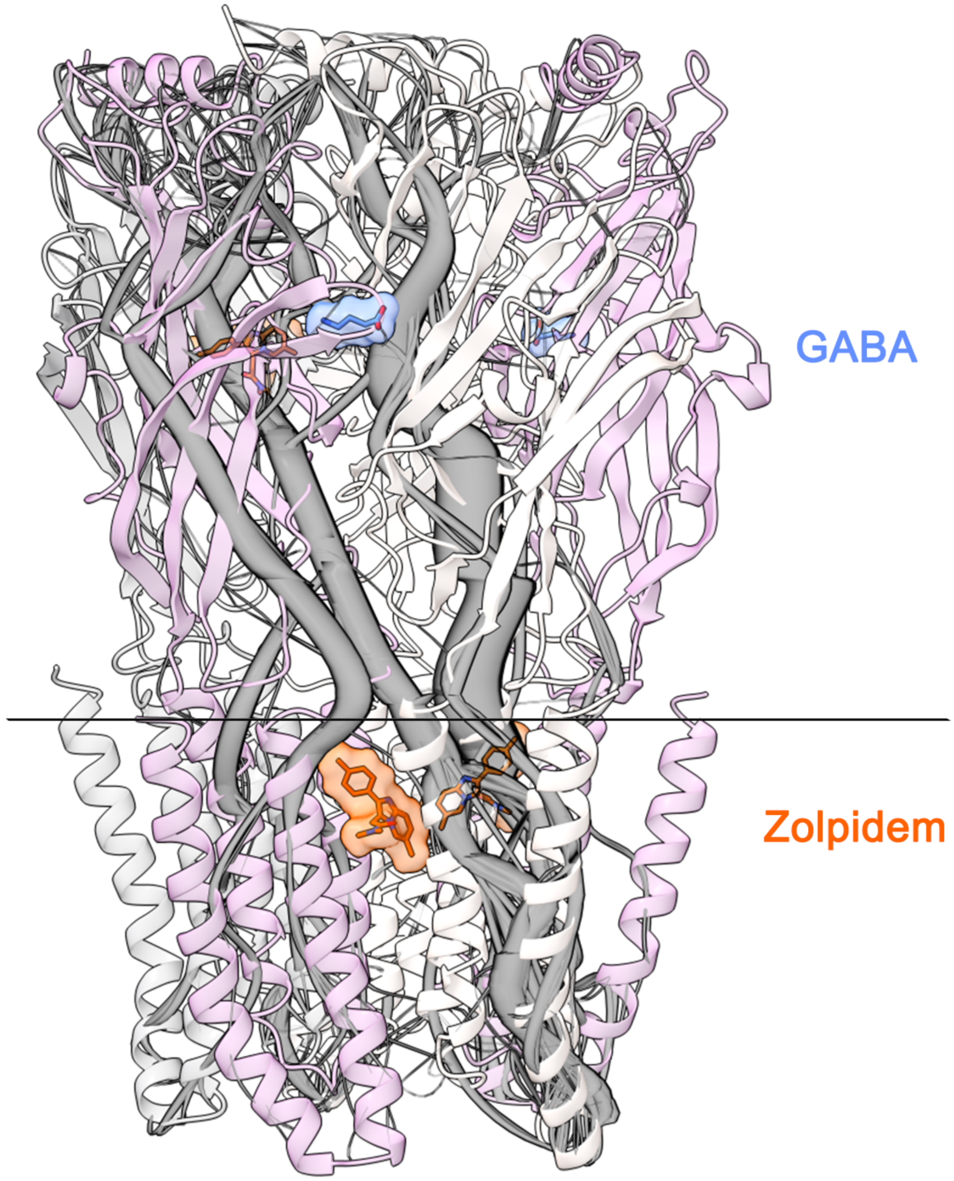

Figure S4: A. Allosteric communication (grey lines) starting in the TMD around the interacting residues of zolpidem (orange) connecting towards the binding pocket of GABA (blue) bound in the ECD.

S1.4 Etomidate

Table S4: Summary of receptor-ligand interactions identified using *Dynophores*.[1–6] Key binding site residues and interaction types are listed for each binding pocket.

**Pocket**	**$\mathbf{GABA_A}$ Residues**	**Interaction Types**
β2A-α1B	M236$^{\alpha 1B}$, L232$^{\alpha 1B}$, M286$^{\beta 1A}$ T262$^{\beta 2A}$, M261$^{\beta 2A}$	Hydrophobic
α1D-β2C	I264$^{\beta 2C}$, T265$^{\alpha 1D}$, V258$^{\beta 2C}$ T237$^{\alpha 2D}$	Hydrophobic

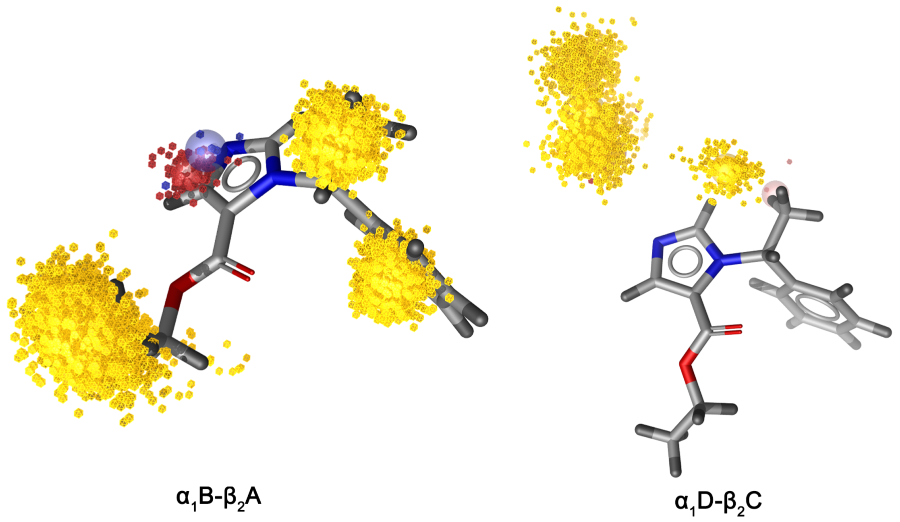

Figure S5: Point cloud representation of the etomidate dynophore. We can see concise point clouds proving the stability of the ligand during the MD simulation. Each point within a cloud represents an interaction in one frame, with green hydrogen bond donors, red hydrogen bond acceptors and yellow hydrophobic contacts.

S1.5 Phenobarbital

Table S5: Summary of receptor-ligand interactions identified using *Dynophores*.[1–6] Key binding site residues and interaction types are listed for each binding pocket.

**Pocket**	**GABA$_\mathbf{A}$ Residues**	**Interaction Types**
γ2E-β2A	V293$^{\gamma 2\mathrm{E}}$, M227$^{\beta 2\mathrm{A}}$, V300$^{\gamma 2\mathrm{E}}$ T277$^{\gamma 2\mathrm{E}}$	Hydrophobic
	L223$^{\beta 2\mathrm{A}}$, S280$^{\gamma 2\mathrm{E}}$, D297$^{\gamma 2\mathrm{E}}$	HBD
	S301$^{\gamma 2\mathrm{E}}$, S280$^{\gamma 2\mathrm{E}}$, T277$^{\gamma 2\mathrm{E}}$ Q224$^{\beta 2\mathrm{A}}$	HBA
	S301$^{\gamma 2\mathrm{E}}$	Aromatic
α1B-β2C	Y294$^{\alpha 1\mathrm{B}}$, M227$^{\beta 2\mathrm{A}}$, I290$^{\alpha 1\mathrm{B}}$ S270$^{\alpha 1\mathrm{B}}$	Hydrophobic
	S270$^{\alpha 1\mathrm{B}}$	HBD
	S270$^{\alpha 1\mathrm{B}}$	HBA
	A291$^{\alpha 1\mathrm{B}}$	Aromatic

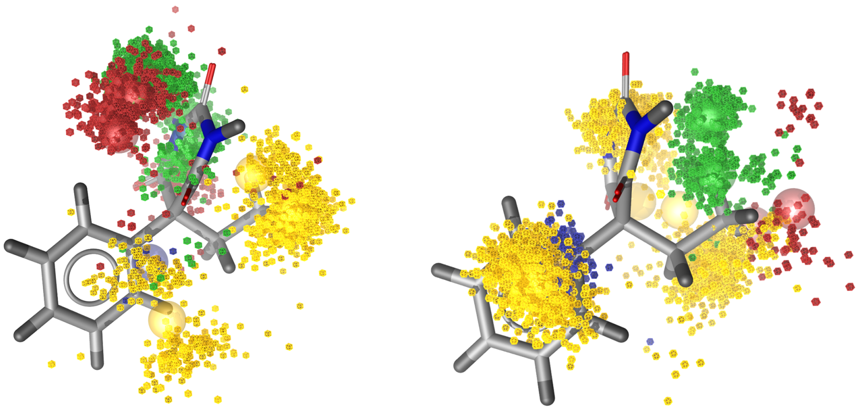

γ_2E-β_2A α_1B-β_2A

Figure S6: Point cloud representation of the phenobarbital dynophore. We can see concise point clouds proving the stability of the ligand during the MD simulation. Each point within a cloud represents an interaction in one frame, with green hydrogen bond donors, red hydrogen bond acceptors and yellow hydrophobic contacts.

S2 Diazepam GABA probe dependence

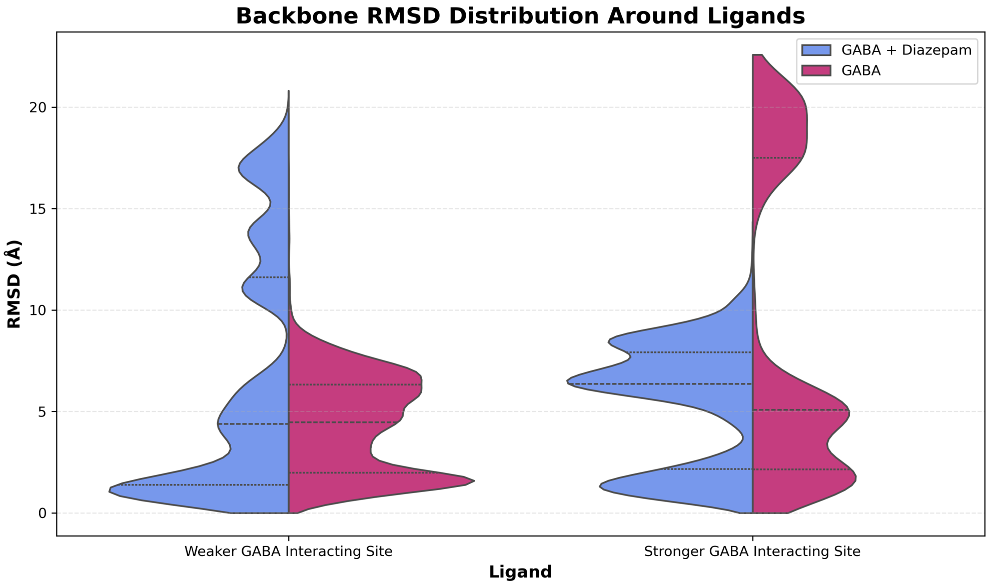

Figure S7: Backbone RMSD distribution around GABA binding sites. Split violin plots show backbone flexibility of residues within 5 Å of two GABA binding sites with (pink) and without (blue) diazepam. Data pooled from three MD replicates. Diazepam reduces flexibility at the weaker interacting site but not the stronger site, indicating site-specific allosteric stabilization.

S3 Zolpidem GABA probe dependence

Given that Z-drugs represent the only other major drug class exhibiting strict GABA dependence, we next investigated zolpidem binding. Strikingly, zolpidem crystallized exclusively at the two TMD positions corresponding precisely to the diazepam sites that showed direct allosteric coupling to GABA, as well as at the ECD position occupied by diazepam. Zolpidem induced similar GABA interaction enhancements as diazepam. At the initially strong-binding site, GABA showed increased HBD interactions with Y157 (66.7%, 59.2%, 70.7%), enhanced HBA interactions with T130 (19%, 2.1%, 10.6%) and R67 (18.9%, 17.8%, 12.4%), and negative ionizable interactions with T202 (12.2%, 5.8%, 9.6%). The weak-binding GABA molecule displayed enhanced HBD interactions with E155 (78.5%, 92.2%, 22.4%) and S156 (21.4%, 21.1%, 16.6%), along with increased HBA interactions with T202 (75.7%, 67.6%, 54%) and R67 (29.9%, 11.5%, 23%).

MDPath analysis revealed a similar allosteric communication pattern for zolpidem compared to diazepam. In all but one replica, allosteric pathways originating from TMD-bound zolpidem connected directly to the ECD-bound GABA molecules. However, the nature of stabilization differed subtly: diazepam occupies three TMD sites compared to two for zolpidem, leading to a narrowing of the previously occupied third binding pocket.

S4 Explainable AI of deep-learned $GABA_A$ State Transition Pathways

To understand and explain the driving differences for the model, which allowed for the learning of the different state transitions, we employed a gradient based attribution on the 10000 input features. Robust agreement between standard gradient and integrated gradient attribution methods, validated the stability of feature rankings. Integrated gradients consistently yielded ∼38-fold higher attribution scores than standard gradients, reflecting their comprehensive path-integration methodology.

Feature attribution analysis using gradient-based methods demonstrated that the DeepTDA model's state classification relies on a highly selective subset of Cα–Cα distances in the case of the initial GABA model. Among the 10,000 ANOVA-selected features, the top 100 distances achieved combined importance scores of 0.0088–0.017, markedly exceeding the population mean of 0.0021 ± 0.0015. This concentration of predictive power indicates that the model extracted a compact, interpretable representation centered on specific structural motifs rather than distributing information diffusely across all input features.

Comparing this initial GABA model to models created with diazepam and phenobarbital also revealed differences in the estimated slowest kinetic process connecting the different receptor states. The GABA model emphasizes the most intracellular part of the TM3 of the $\gamma 2$ - $\alpha 1$ and $\gamma 2$ - $\beta 2$ distance, which is directly below the spots occupied by the allosteric modulators that prevent leaving the open state.

This pattern is largely matched by the phenobarbital + GABA model, which predicted similar dynamics with slight reweighting between ECD and TM features. The diazepam + GABA model shifted considerably. It favored features directly above and between the diazepam molecules in the TMD. Interestingly, it also mapped distances within the binding pocket of the weaker-binding GABA molecule.

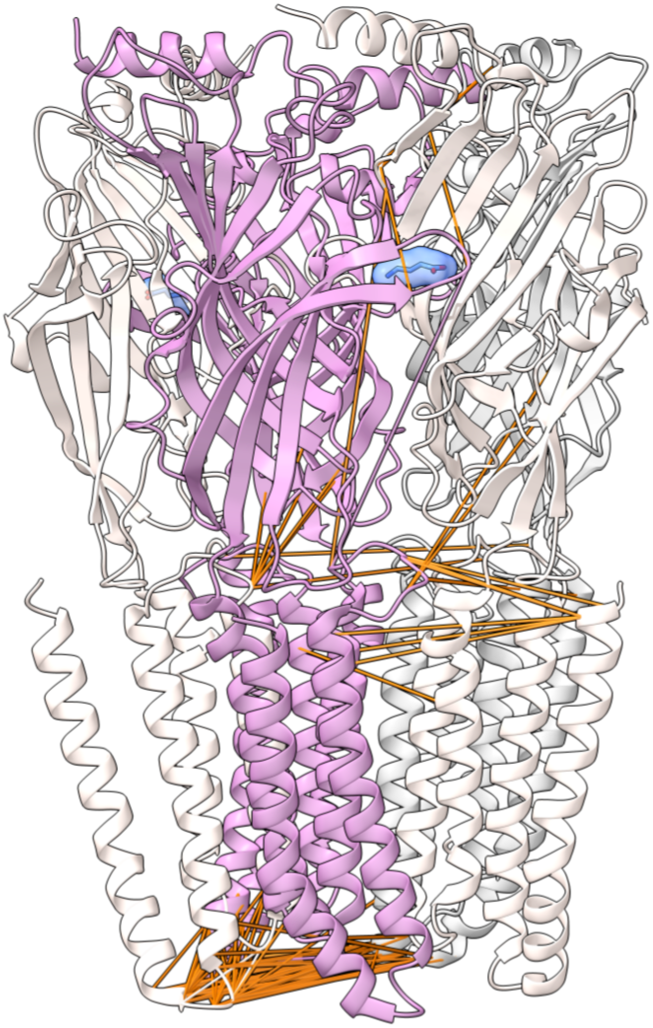

Figure S8: The $GABA_A$ receptor showcased translucently with GABA-bound in the ECD domain (blue). Orange lines indicate the top 100 distances identified by gradient-based attribution of the deepTDA model.

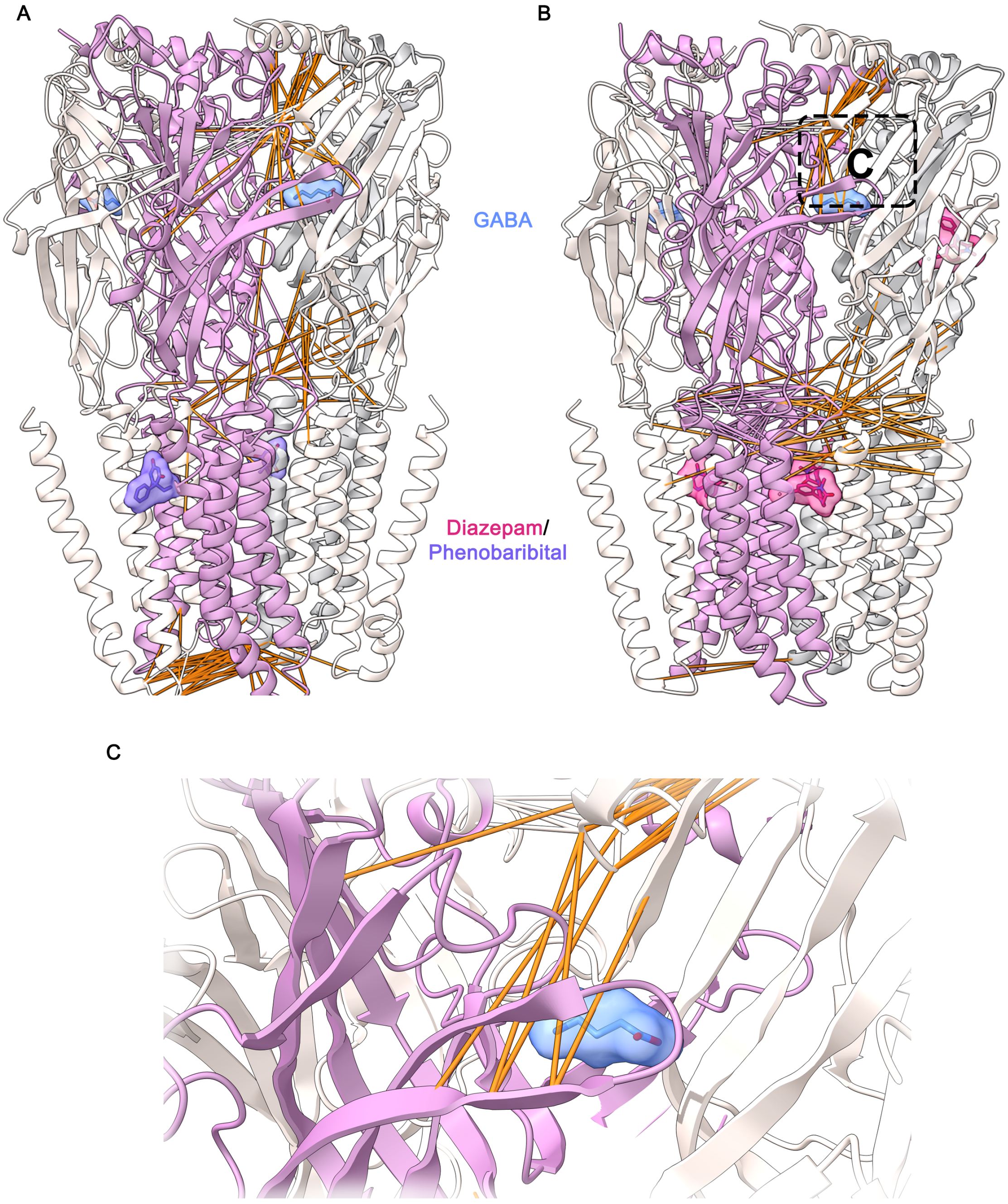

Figure S9: **A** The receptor bound with GABA (blue) in the ECD domain and phenobarbital (purple) binding in the TMD domain. Orange lines indicate the distances identified as the top 100 features predicting the theoretical state transitions by the gradient based attribution. **B** The receptor bound with GABA (blue) in the ECD domain and diazepam (red). Orange lines indicate the distances identified as the top 100 features predicting the theoretical state transitions by the gradient based attribution. **C** A zoom in of the weaker binding GABA molecule showcasing importance for the state transition.

S5 DeepTDA Details

Hyperparameter Optimization

Table S6: Summary of the optimal hyperparameters and final loss for each deepTDA model.

Open	Layers	Learning rate	Weight decay	Activation	Final loss
GABA	512, 1152, 1408, 1152	2.43×10^{-4}	2.71×10^{-5}	ReLU	0.062
GABA + diazepam	1280, 128	1.06×10^{-4}	3×10^{-6}	ELU	0.048
GABA + phenobarbital	2048, 1536, 1280	1.31×10^{-4}	5.4×10^{-5}	ReLU	0.098

S6 H101 mutations

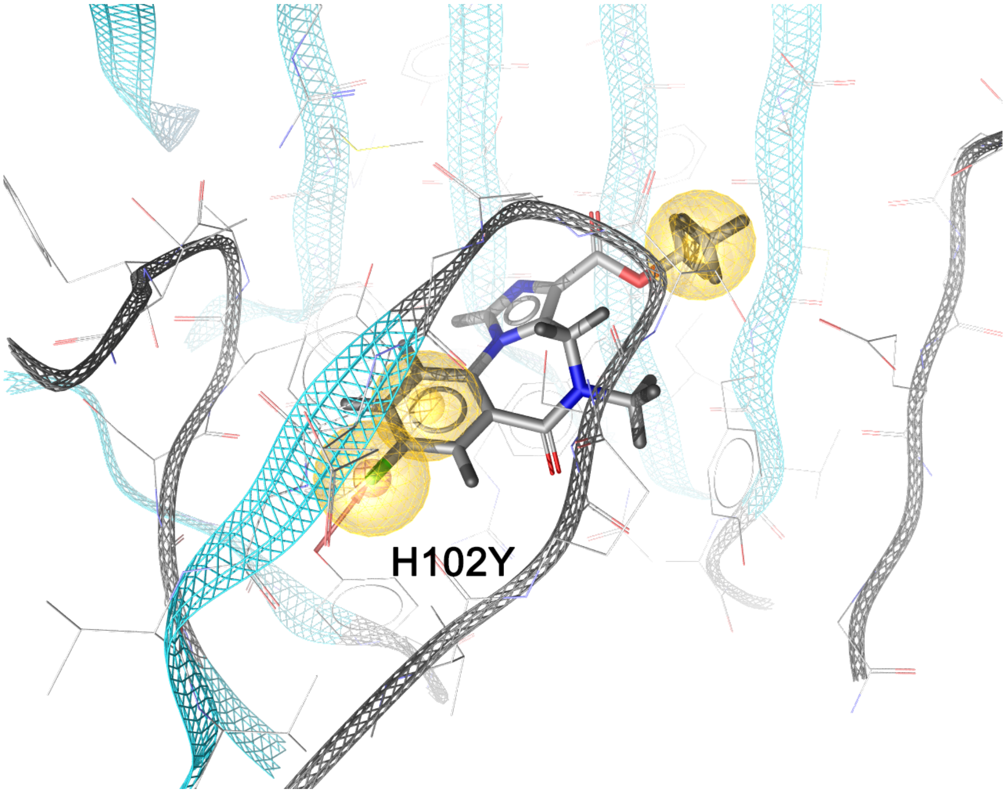

Figure S10: The mutation was introduced in MOE and subsequently energy-minimized using the AMBER14:ETH force field. A pharmacophore model was generated in LigandScout to illustrate key flumazenil–receptor interactions. Yellow spheres indicate hydrophobic contact points, while the pink arrow denotes a halogen-bond interaction. Overall, the mutation enhances interactions with the α1 domain, which in turn reduces the potential for interactions with the γ2 domain via the imidazole ring and its ethyl-ester substituent. As a result, flumazenil can act as a partial agonist by adopting a conformation similar to that of benzodiazepines in the non-mutated A-loop.

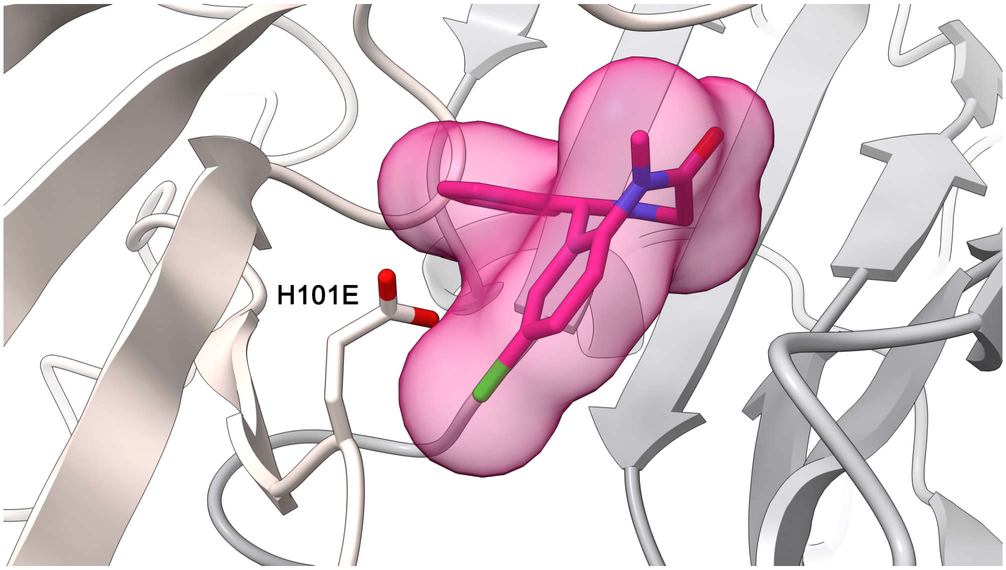

Figure S11: The mutation was introduced in MOE and subsequently energy-minimized using the AMBER14:ETH force field, and the results were visualized by overlaying the original benzodiazepine binding pose with the mutated pocket. The H101E mutation effectively occludes this region: the charged side chain occupies the space that would normally accommodate the hydrophobic benzodiazepine substituents. This explains how A-loop mutations prevent binding of the benzodiazepine scaffold.

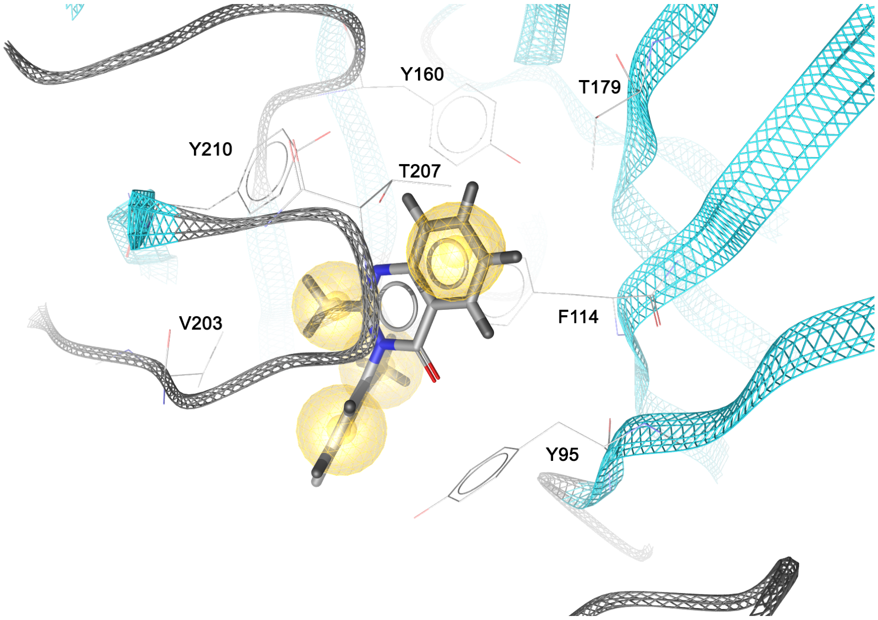

Figure S12: Methaqualone binding in the non-mutated pocket (PDB: 8VQY[7]). Pharmacophores were generated using Ligandscout following energy minimization. Yellow spheres indicate hydrophobic contacts.

S7 Flumazenil allosteric communication

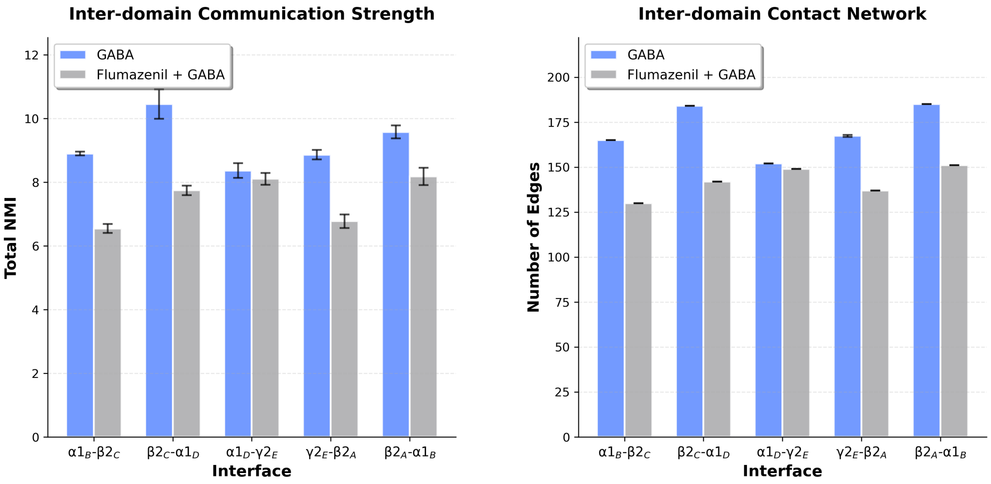

Figure S13: Inter-domain communication strength comparing GABA with GABA and flumazenil. NMI information is pooled from all edges crossing the subunit borders (left). Inter-domain contact network, as a number of edges that describe the NMI information (right).

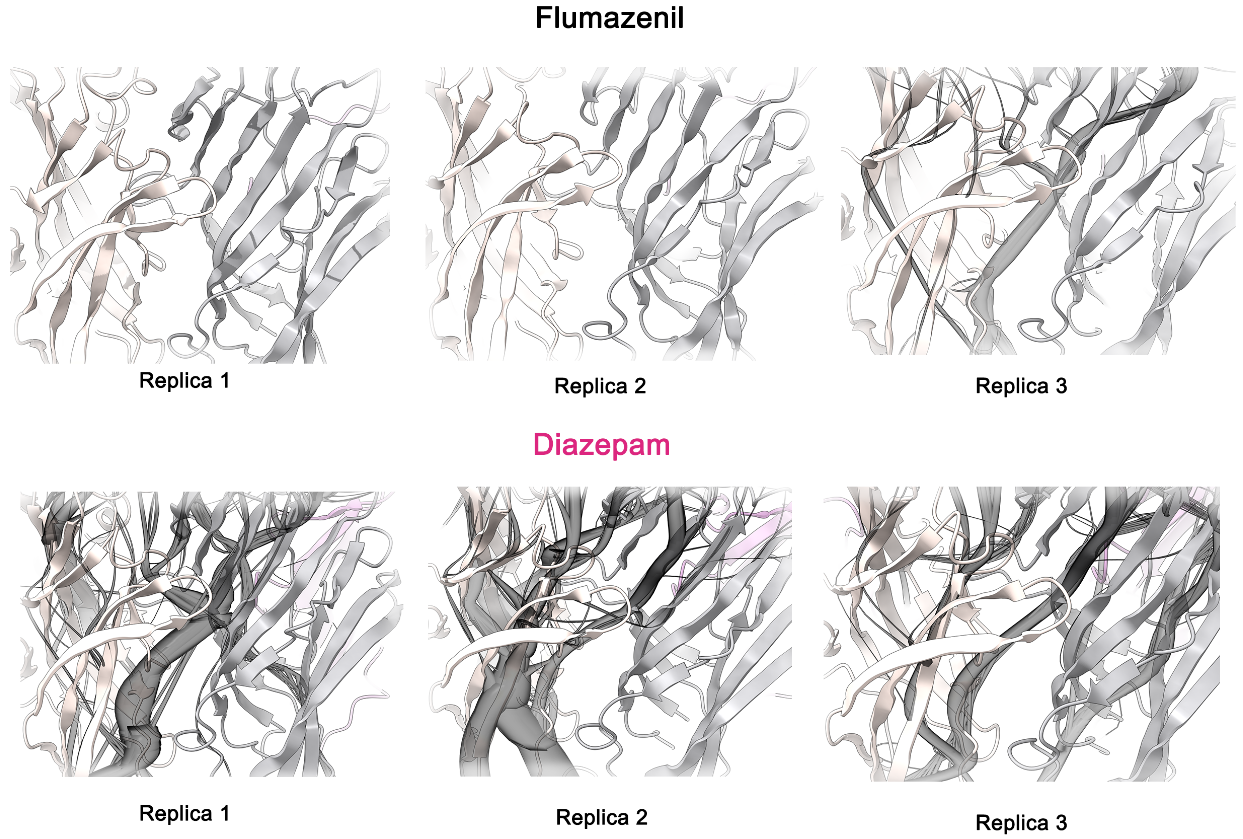

Figure S14: Allosteric communication pathways computed with MDPath for all flumazenil- and diazepam-bound replicas. Clusters identified as crossing the ECD modulator binding pocket are shown as dark paths. In the flumazenil-bound replicas, only weak, fractional communication was observed in a single replica, with no detectable communication in the remaining replicas. By contrast, strong allosteric communication was consistently observed across all diazepam-bound replicas.

S8 Numbering and alignment

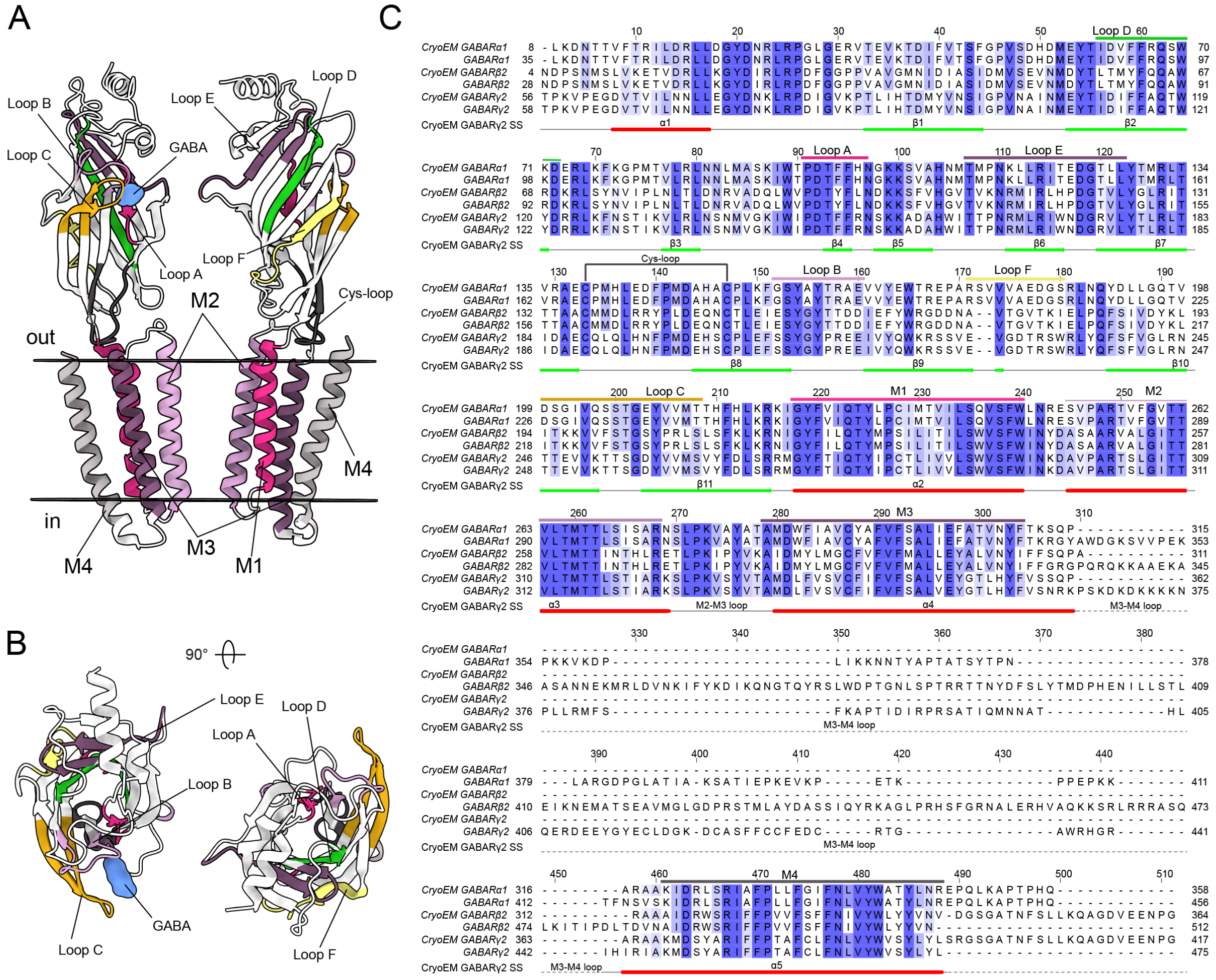

Figure S15: **Structural and sequence features of the $GABA_A$ receptor. (A–B)** Cryo-EM structure of the $GABA_A$ receptor (PDB: 6X3X), shown in cartoon representation with GABA depicted as a surface. Subunits $\beta2$ (chain A) and $\gamma2$ (chain E) are highlighted and colored according to regions involved in the agonist-binding site and transmembrane domain. Key structural elements, including loops A–F, the Cys-loop, and transmembrane helices (M1–M4), are indicated. Panel B shows a 90° rotated view. **(C)** Multiple sequence alignment of human $GABA_A$ receptor $\alpha1$, $\beta2$, and $\gamma2$ subunits (UniProt: P14867, P28472, P18507), labeled as GABAR$\alpha1$, GABAR$\beta2$, and GABAR$\gamma2$, together with the corresponding cryo-EM construct sequences (CryoEM GABAR$\alpha1$, CryoEM GABAR$\beta2$, and CryoEM GABAR$\gamma2$). The alignment was generated using Clustal Omega,[8,9] visualized in Jalview,[10] and colored by sequence identity (blue scale). Secondary structure elements derived from the $GABA_A$ $\gamma2$ subunit are mapped above the alignment, with α-helices shown in red, β-strands as green arrows, and coils as gray lines; unresolved regions in the cryo-EM structure are indicated by dashed lines.

S9 Validation and Convergence of MD Simulations

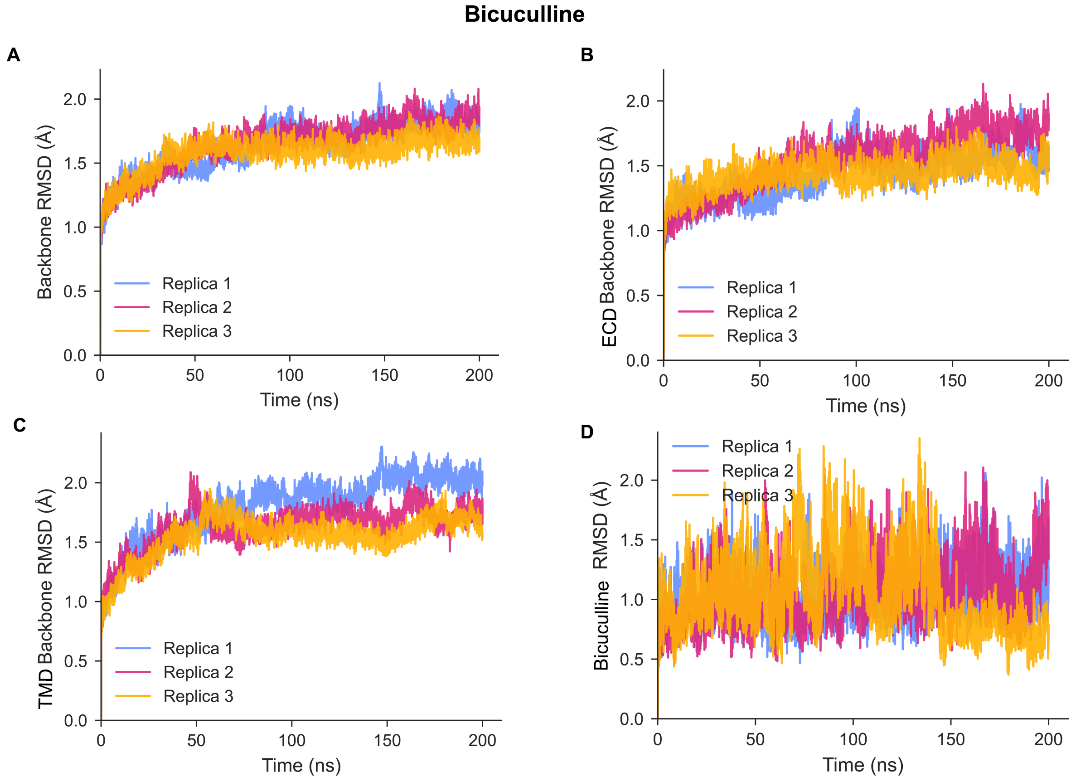

Figure S16: Backbone RMSD time courses assessing system stability over the course of the MD simulations. **(A)** Whole-protein backbone. **(B)** Backbone of the extracellular domain (ECD), defined as all residues from the N-terminus up to the M1 helix. **(C)** Backbone of the transmembrane domain (TMD), defined as all residues outside the ECD. **(D)** Heavy-atom RMSD of bicuculline.

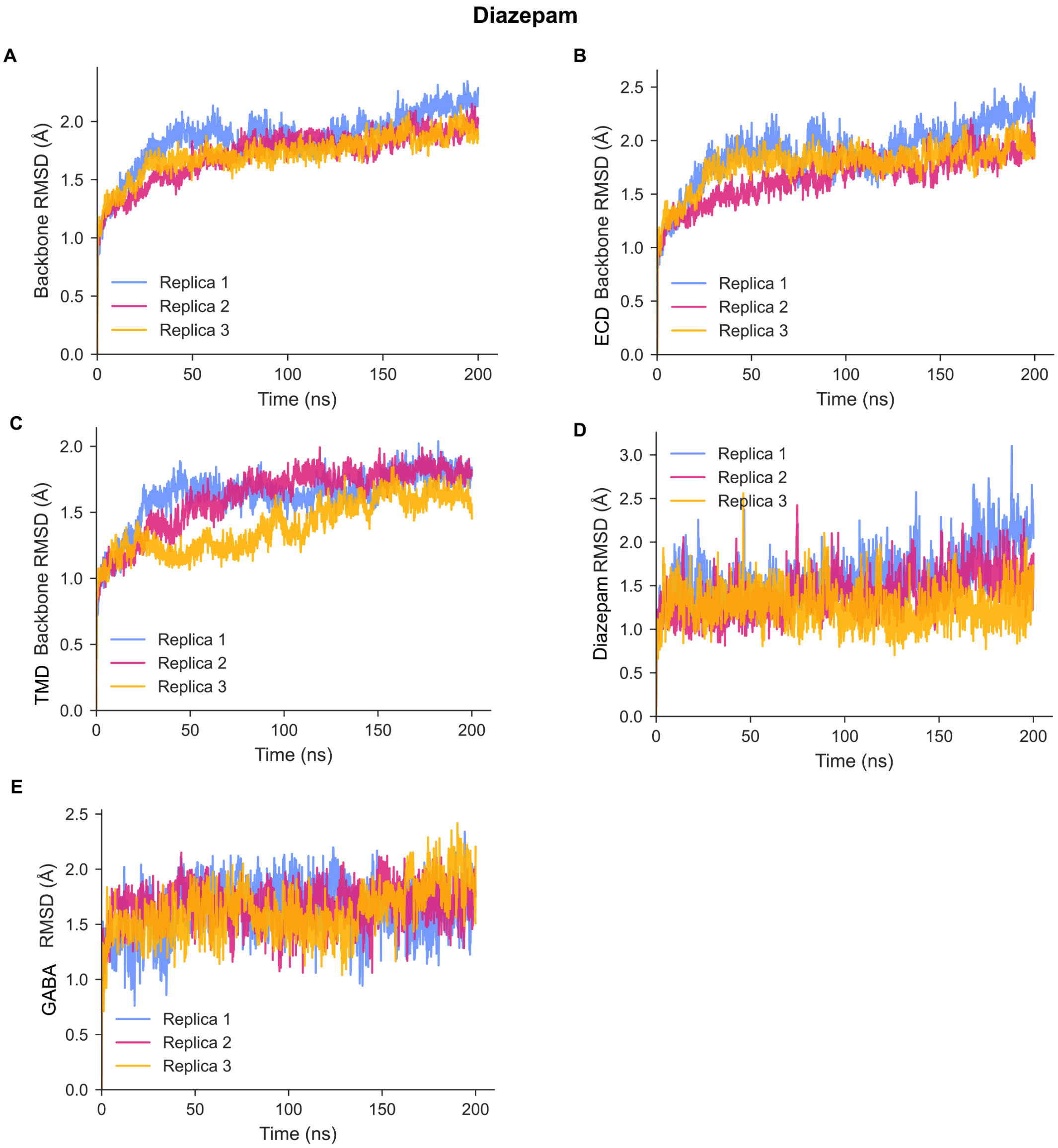

Figure S17: Backbone RMSD time courses assessing system stability over the course of the MD simulations. **(A)** Whole-protein backbone. **(B)** Backbone of the extracellular domain (ECD), defined as all residues from the N-terminus up to the M1 helix. **(C)** Backbone of the transmembrane domain (TMD), defined as all residues outside the ECD. **(D)** Heavy-atom RMSD of diazepam and **E** the ECD bound GABA.

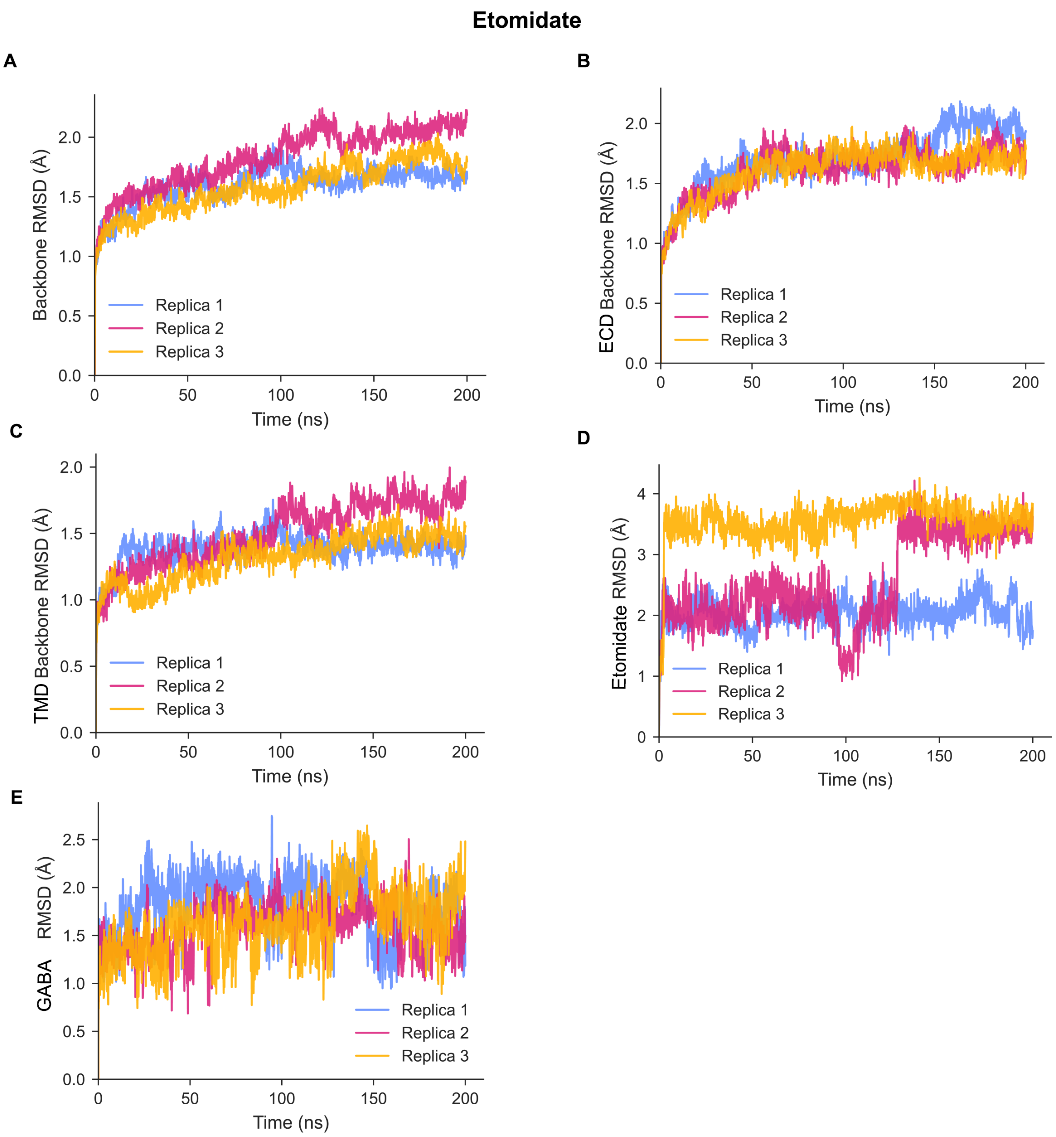

Figure S18: Backbone RMSD time courses assessing system stability over the course of the MD simulations. **(A)** Whole-protein backbone. **(B)** Backbone of the extracellular domain (ECD), defined as all residues from the N-terminus up to the M1 helix. **(C)** Backbone of the transmembrane domain (TMD), defined as all residues outside the ECD. **(D)** Heavy-atom RMSD of etomidate and **E** the ECD bound GABA.

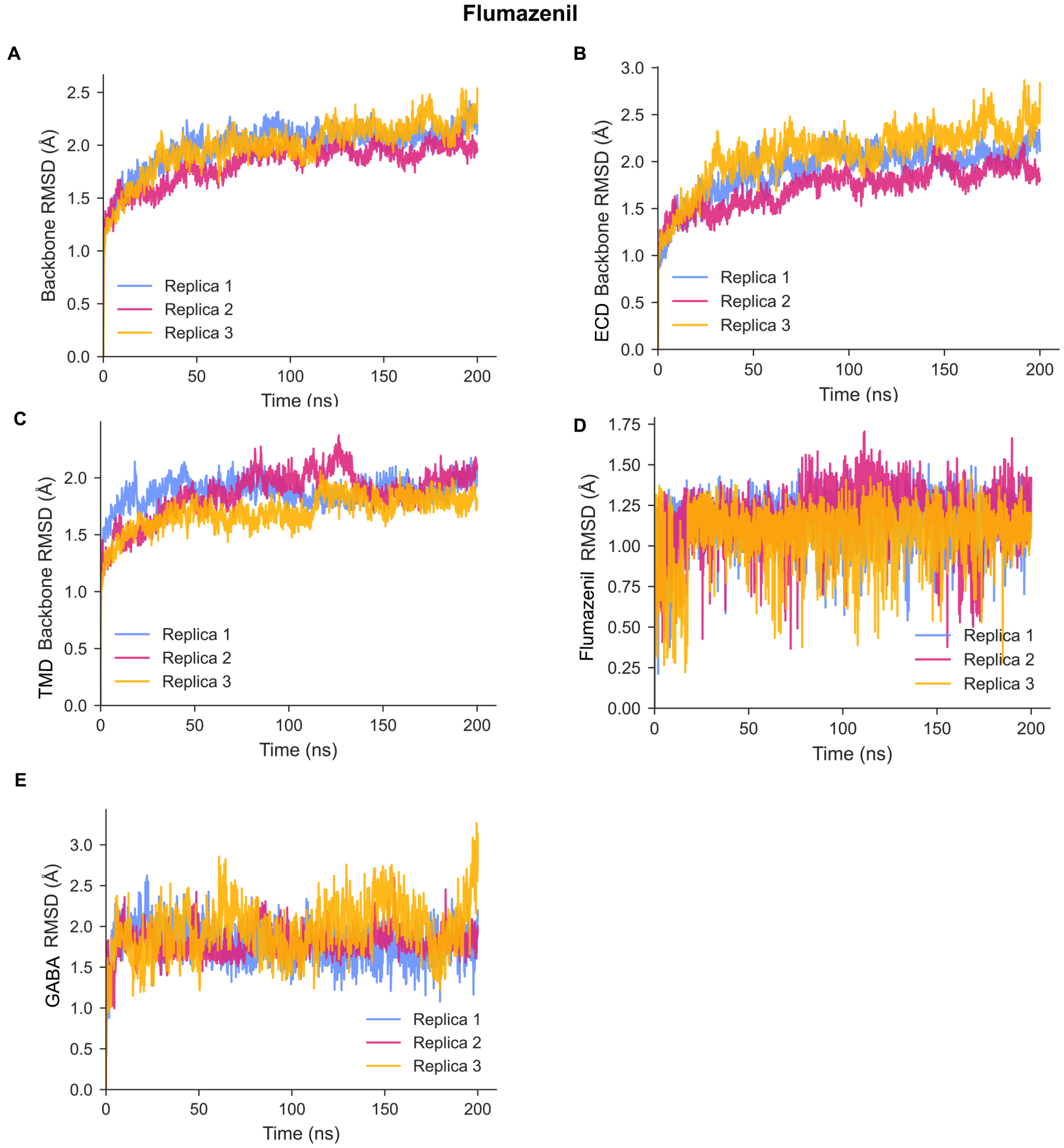

Figure S19: Backbone RMSD time courses assessing system stability over the course of the MD simulations. **(A)** Whole-protein backbone. **(B)** Backbone of the extracellular domain (ECD), defined as all residues from the N-terminus up to the M1 helix. **(C)** Backbone of the transmembrane domain (TMD), defined as all residues outside the ECD. **(D)** Heavy-atom RMSD of flumazenil and **E** the ECD bound GABA.

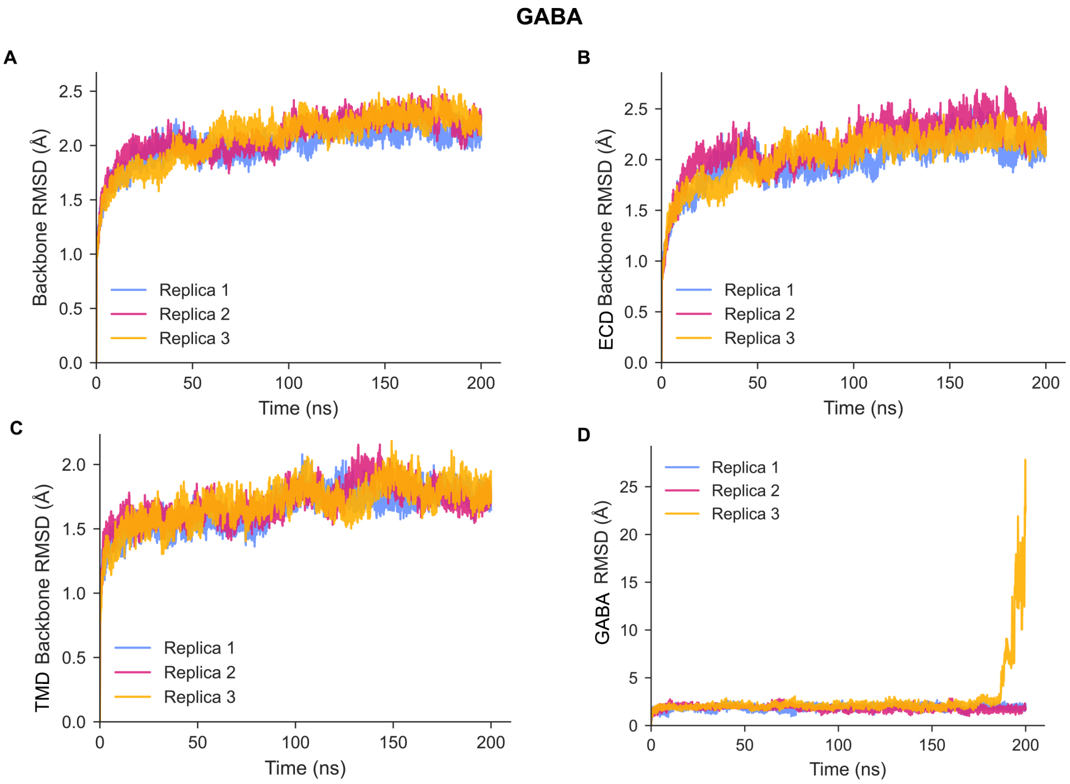

Figure S20: Backbone RMSD time courses assessing system stability over the course of the MD simulations. **(A)** Whole-protein backbone. **(B)** Backbone of the extracellular domain (ECD), defined as all residues from the N-terminus up to the M1 helix. **(C)** Backbone of the transmembrane domain (TMD), defined as all residues outside the ECD. **(D)** Heavy-atom RMSD of GABA. The unbinding event of GABA in replica 3 is further discussed in section "The mechanism of GABA probe dependence".

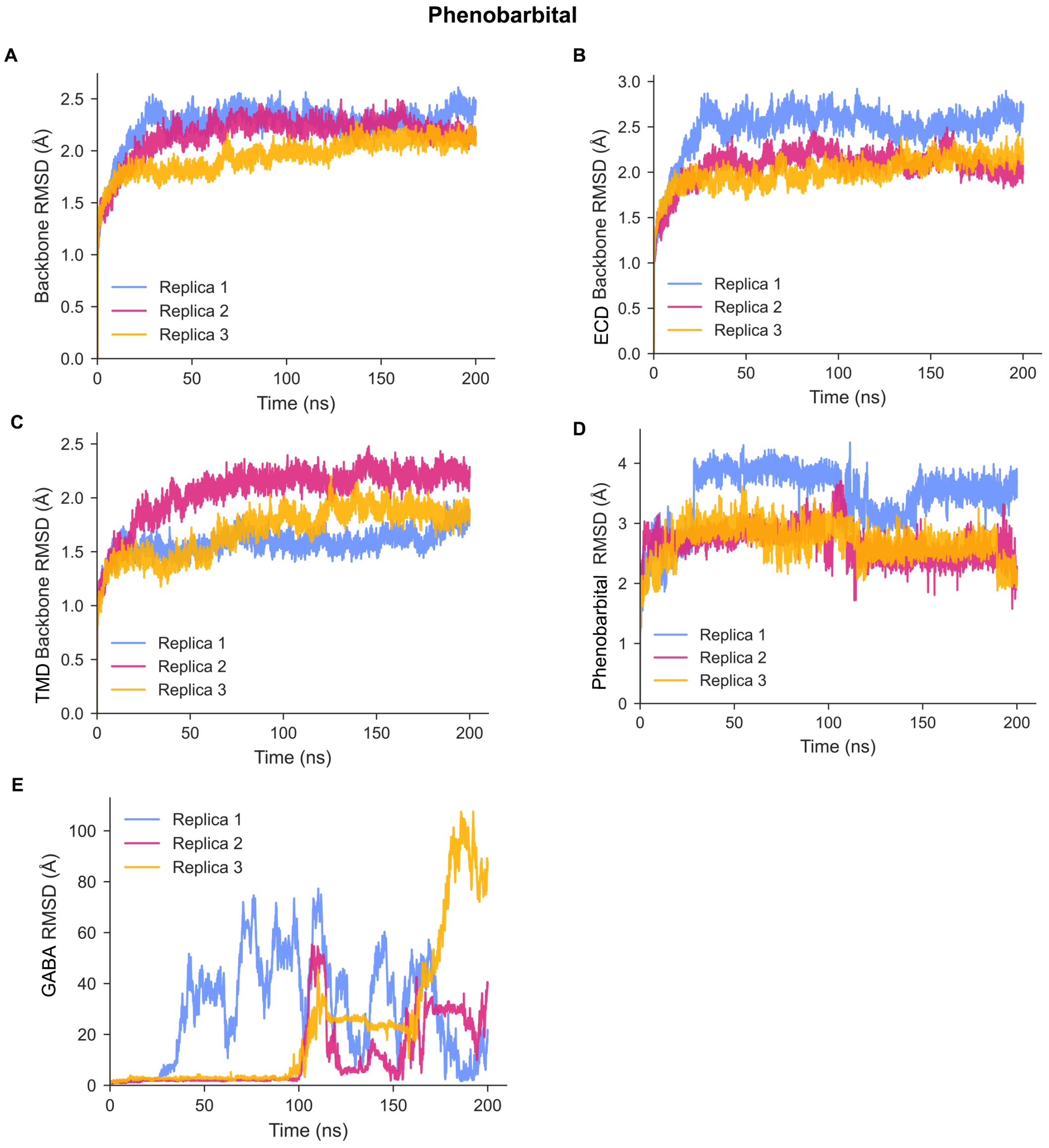

Figure S21: Backbone RMSD time courses assessing system stability over the course of the MD simulations. **(A)** Whole-protein backbone. **(B)** Backbone of the extracellular domain (ECD), defined as all residues from the N-terminus up to the M1 helix. **(C)** Backbone of the transmembrane domain (TMD), defined as all residues outside the ECD. **(D)** Heavy-atom RMSD of phenobarbital and **E** the ECD bound GABA.

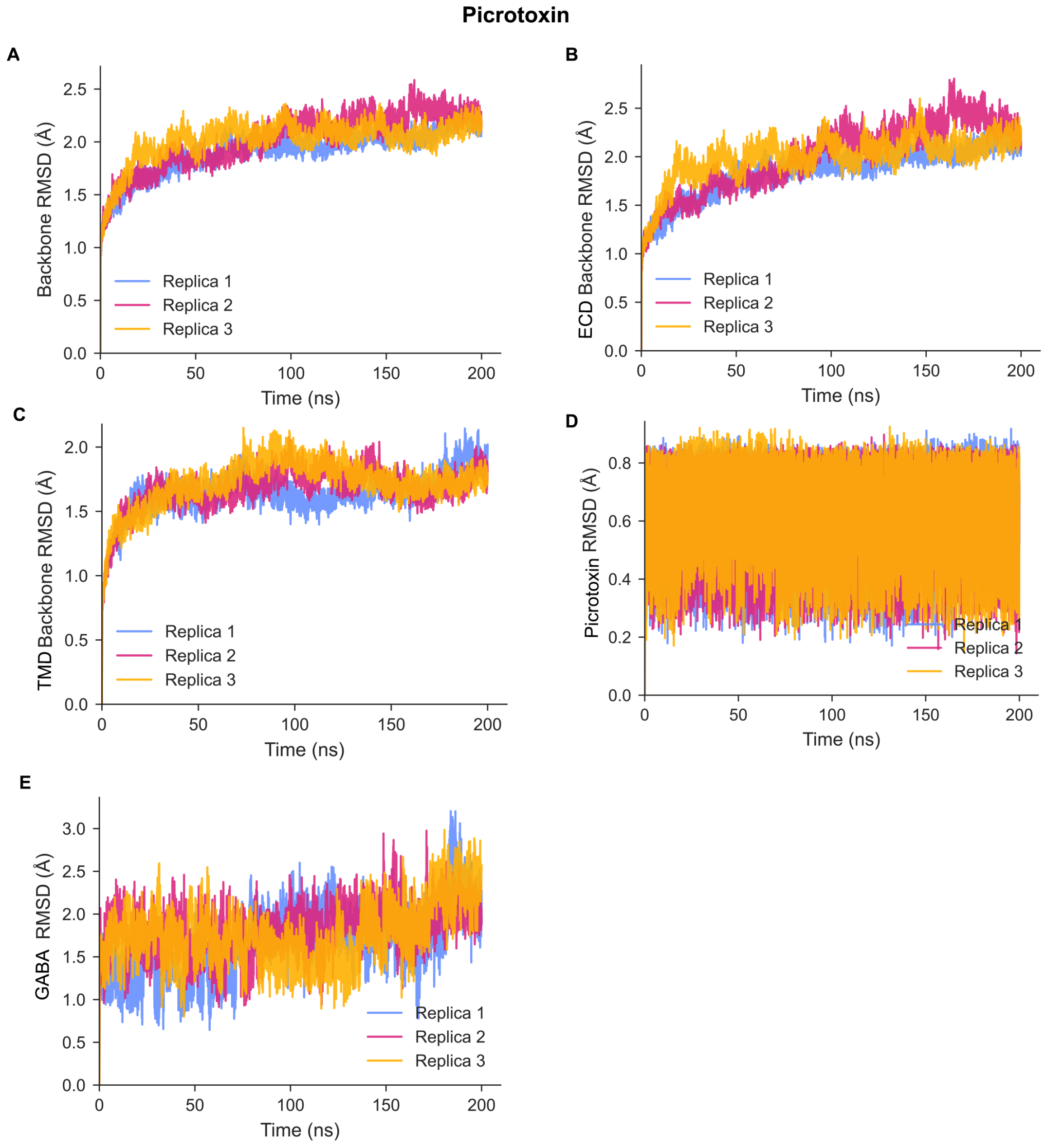

Figure S22: Backbone RMSD time courses assessing system stability over the course of the MD simulations. **(A)** Whole-protein backbone. **(B)** Backbone of the extracellular domain (ECD), defined as all residues from the N-terminus up to the M1 helix. **(C)** Backbone of the transmembrane domain (TMD), defined as all residues outside the ECD. **(D)** Heavy-atom RMSD of picrotoxin and **E** the ECD bound GABA.

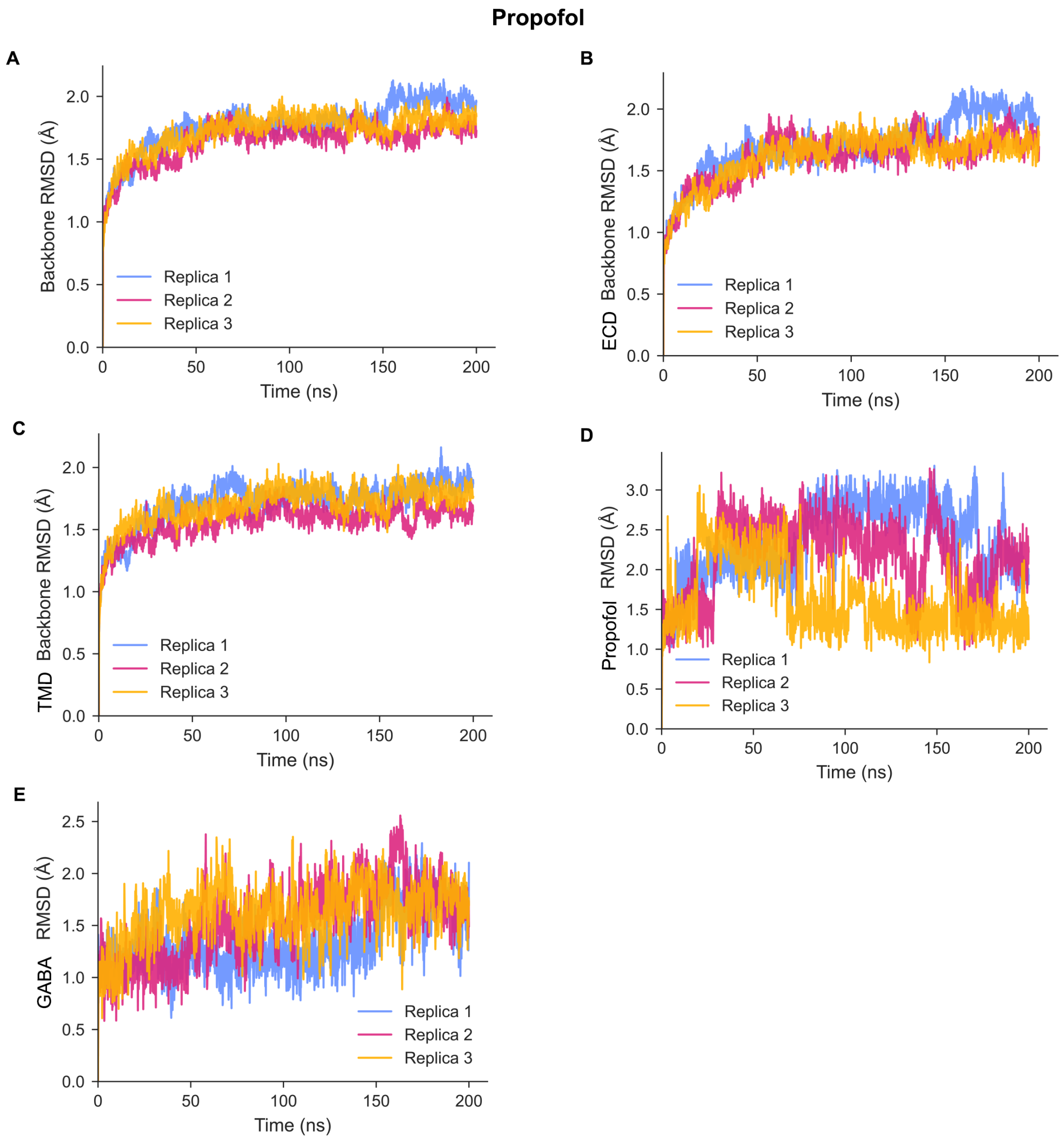

Figure S23: Backbone RMSD time courses assessing system stability over the course of the MD simulations. **(A)** Whole-protein backbone. **(B)** Backbone of the extracellular domain (ECD), defined as all residues from the N-terminus up to the M1 helix. **(C)** Backbone of the transmembrane domain (TMD), defined as all residues outside the ECD. **(D)** Heavy-atom RMSD of propofol and **E** the ECD bound GABA.

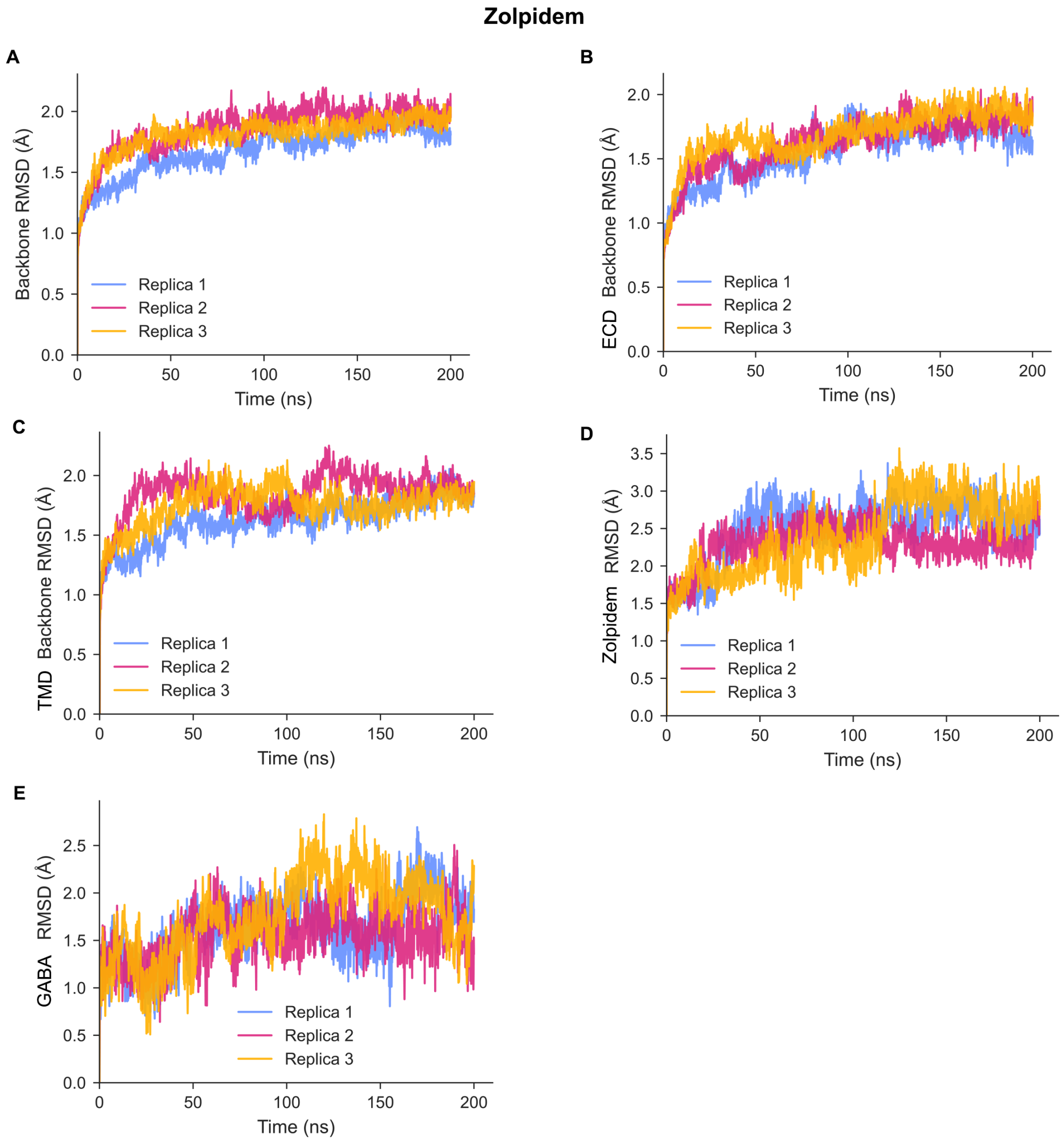

Figure S24: Backbone RMSD time courses assessing system stability over the course of the MD simulations. **(A)** Whole-protein backbone. **(B)** Backbone of the extracellular domain (ECD), defined as all residues from the N-terminus up to the M1 helix. **(C)** Backbone of the transmembrane domain (TMD), defined as all residues outside the ECD. **(D)** Heavy-atom RMSD of zolpidem and **E** the ECD bound GABA.

List of Supplementary Information Figures

S1 Point cloud representation of the diazepam dynophore. We can see concise point clouds proving the stability of the ligand during the MD simulation. Each point within a cloud represents an interaction in one frame, with blue indicating positive ionizable interaction, red hydrogen bond acceptors and yellow hydrophobic contacts. 5

S2 Point cloud representation of the propofol dynophore. We can see concise point clouds proving the stability of the ligand during the MD simulation. Each point within a cloud represents an interaction in one frame, with green hydrogen bond donors, red hydrogen bond acceptors and yellow hydrophobic contacts. 6

S3 Point cloud representation of the zolpidem dynophore. We can see concise point clouds proving the stability of the ligand during the MD simulation. Each point within a cloud represents an interaction in one frame, with blue indicating positive ionizable interaction, red hydrogen bond acceptors and yellow hydrophobic contacts. 8

S4 A. Allosteric communication (grey lines) starting in the TMD around the interacting residues of zolpidem (orange) connecting towards the binding pocket of GABA (blue) bound in the ECD. 9

S5 Point cloud representation of the etomidate dynophore. We can see concise point clouds proving the stability of the ligand during the MD simulation. Each point within a cloud represents an interaction in one frame, with green hydrogen bond donors, red hydrogen bond acceptors and yellow hydrophobic contacts. 10

S6 Point cloud representation of the phenobarbital dynophore. We can see concise point clouds proving the stability of the ligand during the MD simulation. Each point within a cloud represents an interaction in one frame, with green hydrogen bond donors, red hydrogen bond acceptors and yellow hydrophobic contacts. 12

S7 Backbone RMSD distribution around GABA binding sites. Split violin plots show backbone flexibility of residues within 5 Å of two GABA binding sites with (pink) and without (blue) diazepam. Data pooled from three MD replicates. Diazepam reduces flexibility at the weaker interacting site but not the stronger site, indicating site-specific allosteric stabilization. 13
S8 The $GABA_A$ receptor showcased translucently with GABA-bound in the ECD domain (blue). Orange lines indicate the top 100 distances identified by gradient-based attribution of the deepTDA model. 16
S9 **A** The receptor bound with GABA (blue) in the ECD domain and phenobarbital (purple) binding in the TMD domain. Orange lines indicate the distances identified as the top 100 features predicting the theoretical state transitions by the gradient based attribution. **B** The receptor bound with GABA (blue) in the ECD domain and diazepam (red). Orange lines indicate the distances identified as the top 100 features predicting the theoretical state transitions by the gradient based attribution. **C** A zoom in of the weaker binding GABA molecule showcasing importance for the state transition. 17
S10 The mutation was introduced in MOE and subsequently energy-minimized using the AMBER14:ETH force field. A pharmacophore model was generated in LigandScout to illustrate key flumazenil–receptor interactions. Yellow spheres indicate hydrophobic contact points, while the pink arrow denotes a halogen-bond interaction. Overall, the mutation enhances interactions with the α1 domain, which in turn reduces the potential for interactions with the γ2 domain via the imidazole ring and its ethyl-ester substituent. As a result, flumazenil can act as a partial agonist by adopting a conformation similar to that of benzodiazepines in the non-mutated A-loop. 19

S11 The mutation was introduced in MOE and subsequently energy-minimized using the AMBER14:ETH force field, and the results were visualized by overlaying the original benzodiazepine binding pose with the mutated pocket. The H101E mutation effectively occludes this region: the charged side chain occupies the space that would normally accommodate the hydrophobic benzodiazepine substituents. This explains how A-loop mutations prevent binding of the benzodiazepine scaffold. 20

S12 Methaqualone binding in the non-mutated pocket (PDB: 8VQY[7]). Pharmacophores were generated using Ligandscout following energy minimization. Yellow spheres indicate hydrophobic contacts. 20

S13 Inter-domain communication strength comparing GABA with GABA and flumazenil. NMI information is pooled from all edges crossing the subunit borders (left). Inter-domain contact network, as a number of edges that describe the NMI information (right). 21

S14 Allosteric communication pathways computed with MDPath for all flumazenil- and diazepam-bound replicas. Clusters identified as crossing the ECD modulator binding pocket are shown as dark paths. In the flumazenil-bound replicas, only weak, fractional communication was observed in a single replica, with no detectable communication in the remaining replicas. By contrast, strong allosteric communication was consistently observed across all diazepam-bound replicas. 22

S15 **Structural and sequence features of the GABA$_\mathbf{A}$ receptor. (A–B)** Cryo-EM structure of the GABA$_A$ receptor (PDB: 6X3X), shown in cartoon representation with GABA depicted as a surface. Subunits β2 (chain A) and γ2 (chain E) are highlighted and colored according to regions involved in the agonist-binding site and transmembrane domain. Key structural elements, including loops A–F, the Cys-loop, and transmembrane helices (M1–M4), are indicated. Panel B shows a 90° rotated view. **(C)** Multiple sequence alignment of human GABA$_A$ receptor α1, β2, and γ2 subunits (UniProt: P14867, P28472, P18507), labeled as GABARα1, GABARβ2, and GABARγ2, together with the corresponding cryo-EM construct sequences (CryoEM GABARα1, CryoEM GABARβ2, and CryoEM GABARγ2). The alignment was generated using Clustal Omega,[8,9] visualized in Jalview,[10] and colored by sequence identity (blue scale). Secondary structure elements derived from the GABA$_A$ γ2 subunit are mapped above the alignment, with α-helices shown in red, β-strands as green arrows, and coils as gray lines; unresolved regions in the cryo-EM structure are indicated by dashed lines. 23

S16 Backbone RMSD time courses assessing system stability over the course of the MD simulations. **(A)** Whole-protein backbone. **(B)** Backbone of the extracellular domain (ECD), defined as all residues from the N-terminus up to the M1 helix. **(C)** Backbone of the transmembrane domain (TMD), defined as all residues outside the ECD. **(D)** Heavy-atom RMSD of bicuculline. 24

S17 Backbone RMSD time courses assessing system stability over the course of the MD simulations. **(A)** Whole-protein backbone. **(B)** Backbone of the extracellular domain (ECD), defined as all residues from the N-terminus up to the M1 helix. **(C)** Backbone of the transmembrane domain (TMD), defined as all residues outside the ECD. **(D)** Heavy-atom RMSD of diazepam and **E** the ECD bound GABA. 25

S18 Backbone RMSD time courses assessing system stability over the course of the MD simulations. **(A)** Whole-protein backbone. **(B)** Backbone of the extracellular domain (ECD), defined as all residues from the N-terminus up to the M1 helix. **(C)** Backbone of the transmembrane domain (TMD), defined as all residues outside the ECD. **(D)** Heavy-atom RMSD of etomidate and **E** the ECD bound GABA. 26

S19 Backbone RMSD time courses assessing system stability over the course of the MD simulations. **(A)** Whole-protein backbone. **(B)** Backbone of the extracellular domain (ECD), defined as all residues from the N-terminus up to the M1 helix. **(C)** Backbone of the transmembrane domain (TMD), defined as all residues outside the ECD. **(D)** Heavy-atom RMSD of flumazenil and **E** the ECD bound GABA. 27

S20 Backbone RMSD time courses assessing system stability over the course of the MD simulations. **(A)** Whole-protein backbone. **(B)** Backbone of the extracellular domain (ECD), defined as all residues from the N-terminus up to the M1 helix. **(C)** Backbone of the transmembrane domain (TMD), defined as all residues outside the ECD. **(D)** Heavy-atom RMSD of GABA. The unbinding event of GABA in replica 3 is further discussed in section ”The mechanism of GABA probe dependence”. . . . 28

S21 Backbone RMSD time courses assessing system stability over the course of the MD simulations. **(A)** Whole-protein backbone. **(B)** Backbone of the extracellular domain (ECD), defined as all residues from the N-terminus up to the M1 helix. **(C)** Backbone of the transmembrane domain (TMD), defined as all residues outside the ECD. **(D)** Heavy-atom RMSD of phenobarbital and **E** the ECD bound GABA. . . . 29

S22 Backbone RMSD time courses assessing system stability over the course of the MD simulations. **(A)** Whole-protein backbone. **(B)** Backbone of the extracellular domain (ECD), defined as all residues from the N-terminus up to the M1 helix. **(C)** Backbone of the transmembrane domain (TMD), defined as all residues outside the ECD. **(D)** Heavy-atom RMSD of picrotoxin and **E** the ECD bound GABA. 30

S23 Backbone RMSD time courses assessing system stability over the course of the MD simulations. **(A)** Whole-protein backbone. **(B)** Backbone of the extracellular domain (ECD), defined as all residues from the N-terminus up to the M1 helix. **(C)** Backbone of the transmembrane domain (TMD), defined as all residues outside the ECD. **(D)** Heavy-atom RMSD of propofol and **E** the ECD bound GABA. 31

S24 Backbone RMSD time courses assessing system stability over the course of the MD simulations. **(A)** Whole-protein backbone. **(B)** Backbone of the extracellular domain (ECD), defined as all residues from the N-terminus up to the M1 helix. **(C)** Backbone of the transmembrane domain (TMD), defined as all residues outside the ECD. **(D)** Heavy-atom RMSD of zolpidem and **E** the ECD bound GABA. 32

List of Supplementary Information Tables

S1 Summary of receptor-ligand interactions identified using *Dynophores*.[1–6] Key binding site residues and interaction types are listed for each binding pocket. 4

S2 Summary of receptor-ligand interactions identified using *Dynophores*.[1–6] Key binding site residues and interaction types are listed for each binding pocket. 6

S3 Summary of receptor-ligand interactions identified using *Dynophores*.[1–6] Key binding site residues and interaction types are listed for each binding pocket. 7

S4 Summary of receptor-ligand interactions identified using *Dynophores*.[1–6] Key binding site residues and interaction types are listed for each binding pocket. 10

S5 Summary of receptor-ligand interactions identified using *Dynophores*.[1–6] Key binding site residues and interaction types are listed for each binding pocket. 11

S6 Summary of the optimal hyperparameters and final loss for each deepTDA model. . 18

References

(1) Sydow, D. Dynophores: Novel Dynamic Pharmacophores. 2015.

(2) Bock, A.; Bermudez, M.; Krebs, F.; Matera, C.; Chirinda, B.; Sydow, D.; Dallanoce, C.;

Holzgrabe, U.; De Amici, M.; Lohse, M. J.; Wolber, G.; Mohr, K. Ligand Binding Ensembles Determine Graded Agonist Efficacies at a G Protein-coupled Receptor. *J. Biol. Chem.* **2016**, *291*, 16375–16389.

(3) Wunsch, F.; Nguyen, T. N.; Wolber, G.; Bermudez, M. Structural Determinants of Sphingosine-1-Phosphate Receptor Selectivity. *Arch. Pharm.* **2023**, *356*, 2300387.

(4) Puls, K.; Schmidhammer, H.; Wolber, G.; Spetea, M. Mechanistic Characterization of the Pharmacological Profile of HS-731, a Peripherally Acting Opioid Analgesic, at the Mu-, Delta-, Kappa-Opioid and Nociceptin Receptors. *Molecules* **2022**, *27*, 919.

(5) Schaller, D.; Šribar, D.; Noonan, T.; Deng, L.; Nguyen, T. N.; Pach, S.; Machalz, D.; Bermudez, M.; Wolber, G. Next Generation 3D Pharmacophore Modeling. *Wiley Interdiscip. Rev. Comput. Mol. Sci.* **2020**, *10*, e1468.

(6) Sydow, D.; Wolber, G. dynophores. 2024; `https://github.com/wolberlab/dynophores`, (accessed 2024, October 28).

(7) Chojnacka, W.; Teng, J.; Kim, J. J.; Jensen, A. A.; Hibbs, R. E. Structural insights into GABAA receptor potentiation by Quaalude. *Nature Communications* **2024**, *15*.

(8) Madeira, F.; Madhusoodanan, N.; Lee, J.; Eusebi, A.; Niewielska, A.; Tivey, A. R. N.; Lopez, R.; Butcher, S. The EMBL-EBI Job Dispatcher sequence analysis tools framework in 2024. *Nucleic Acids Research* **2024**, *52*, W521–W525.

(9) Sievers, F.; Wilm, A.; Dineen, D.; Gibson, T. J.; Karplus, K.; Li, W.; Lopez, R.; McWilliam, H.; Remmert, M.; Söding, J.; Thompson, J. D.; Higgins, D. G. Fast, scalable generation of high-quality protein multiple sequence alignments using Clustal Omega. *Molecular Systems Biology* **2011**, *7*.

(10) Waterhouse, A. M.; Procter, J. B.; Martin, D. M. A.; Clamp, M.; Barton, G. J. Jalview Version 2—a multiple sequence alignment editor and analysis workbench. *Bioinformatics* **2009**, *25*, 1189–1191.
